# Within-person reciprocal associations between problematic smartphone use and exercise adherence among university students: a four-wave random-intercept cross-lagged panel study

**DOI:** 10.3389/fpsyg.2026.1907886

**Published:** 2026-08-24

**Authors:** Yihe Gai, Xia Liu, Shuai Sun, Peng Wang, Songbin Wang

**Affiliations:** 1 Department of Physical Education, Hongyi College, Chengdu Technological University, Chengdu, China; 2 Department of Intelligent Manufacturing, Sichuan Yibin Vocational and Technical School, Yibin, China

**Keywords:** exercise adherence, longitudinal study, problematic smartphone use, RI-CLPM, university students

## Abstract

**Background:**

Problematic smartphone use (PSU) and difficulties maintaining exercise-related routines are common among university students, yet the prospective within-person relationship between PSU and self-reported exercise-adherence tendency (EA) remains unclear. Previous cross-sectional and traditional cross-lagged studies cannot cleanly separate stable between-person differences from within-person fluctuations.

**Objective:**

This four-wave longitudinal study examined reciprocal prospective associations between PSU and self-reported EA tendency using a random-intercept cross-lagged panel model (RI-CLPM).

**Methods:**

A total of 1,241 adult university students from six universities in Chengdu, China, provided valid baseline data in a four-wave offline survey conducted from March to December 2025. Longitudinal measurement invariance was evaluated with three subscale-mean indicators per construct. The primary observed-score RI-CLPM used robust maximum likelihood and full-information maximum likelihood. Directional differences were tested using time-specific within-person standardized coefficients and robust Wald tests. Sensitivity analyses included complete-case, partial-equality, multiple-indicator latent, covariate-adjusted, exerciser-only, auxiliary-variable FIML, and delta-adjusted missing-data models.

**Results:**

Scalar invariance was supported at the subscale-indicator level. EA-to-PSU associations were *β* = −0.293, 95% CI [−0.373, −0.213], *β* = −0.185, 95% CI [−0.271, −0.099], and *β* = −0.136, 95% CI [−0.208, −0.064] (all *p* < 0.001). The PSU-to-EA paths were *β* = 0.017, 95% CI [−0.067, 0.102], *β* = 0.027, 95% CI [−0.059, 0.112], and *β* = −0.064, 95% CI [−0.142, 0.014] (all *p* > 0.05). The joint directional test was significant, Wald χ^2^(3) = 59.159, *p* < 0.001; interval-specific differences were significant at T1–T2 and T2–T3 but not T3–T4. The within-person pattern was reproduced across alternative standardizations and sensitivity analyses. The observed-score random-intercept association was not reproduced in the multiple-indicator latent model.

**Conclusion:**

Within-person elevations in self-reported exercise-adherence tendency were prospectively associated with lower subsequent PSU, whereas the reverse paths were not statistically significant. The directional difference was not supported in the final interval, and the observational design does not establish a causal or preventive effect.

## 1 Introduction

University life is a formative period for the development and consolidation of health-related behavioral routines. During this transition, students typically gain greater autonomy over their daily schedules, social activities, digital habits, and exercise behavior. Regular physical activity is widely recognized as beneficial for physical and mental health, and the World Health Organization recommends that adults accumulate sufficient moderate- to vigorous-intensity physical activity each week to obtain health benefits (Bull et al., 2020). Nevertheless, maintaining exercise behavior is often difficult among university students (Babaeer et al., 2022; Favieri et al., 2023; Rivera et al., 2021; Yuan et al., 2024). Previous evidence has shown that many college students do not engage in adequate physical activity, and the challenge is not merely whether students exercise occasionally, but whether they can sustain exercise-related routines over time (Keating et al., 2005; Rhodes and Sui, 2021). In the present study, exercise adherence (EA) denotes a self-reported tendency to maintain exercise-related behavior, encompassing behavioral habit, effortful persistence, and positive exercise-related experience. The score is a behavioral-tendency measure and is not an objective indicator of maintained physical activity or compliance with a prescribed exercise program.

At the same time, smartphones have become deeply embedded in university students’ academic, social, and recreational lives. Smartphones support learning, communication, entertainment, and social connection, but their constant availability may also foster dysregulated patterns of use. Problematic smartphone use (PSU) refers to a maladaptive pattern of smartphone use characterized by impaired control, excessive involvement, and negative consequences in daily functioning (Billieux, 2012; Elhai et al., 2017). In this study, PSU is treated as a continuous behavioral tendency rather than as a clinical diagnosis. This distinction is important because most university students may not meet clinical criteria for behavioral addiction, yet many may still experience varying degrees of difficulty controlling smartphone use (Busch and McCarthy, 2021; Lu et al., 2024). Systematic reviews have linked problematic smartphone use and related digital-behavior problems with adverse outcomes among young people, including symptoms of depression, anxiety, stress, impaired well-being, and academic difficulties (Elhai et al., 2017; Paterna et al., 2024; Shannon et al., 2022; Sohn et al., 2019). Given that university students frequently rely on smartphones for both academic and social purposes, PSU represents a developmentally relevant form of digital-behavior dysregulation.

Theoretically, PSU and EA may be connected because both are embedded in students’ everyday self-regulatory routines. From a behavioral-displacement perspective, excessive smartphone use may compete with exercise for time, attention, and motivational resources. Smartphone use is often sedentary and easily fragmented into repeated checking episodes, which may interrupt planned activities and reduce the likelihood of initiating or maintaining exercise. Empirical work has shown that mobile phone use among college students is associated with sedentary leisure behavior and may interfere with exercise participation (Barkley and Lepp, 2016; Lepp et al., 2013; Zhu et al., 2023). More recent evidence also suggests that reducing smartphone use can be accompanied by increases in physical activity, supporting the possibility that smartphone behavior and movement behavior may be behaviorally linked in daily life (Le Steunf et al., 2024). Related studies in Chinese student and youth samples further indicate that physical activity, anxiety, self-control, and stress processes are intertwined with smartphone addiction or problematic smartphone use (Cao et al., 2023; Lai et al., 2025; Tao et al., 2026; Ye et al., 2025). From the perspective of the Interaction of Person-Affect-Cognition-Execution model, problematic digital behaviors may be maintained through interactions among affective responses, cognitive biases, executive-control processes, and habitual use patterns (Brand et al., 2019). Thus, when a student’s smartphone use becomes more dysregulated than usual, exercise plans may be more easily delayed, interrupted, or abandoned.

A complementary compositional perspective views daily behaviors as parts of a finite time budget. Time allocated to one activity has meaning relative to time allocated to other activities because an increase in one component necessarily corresponds to a decrease in at least one other component. Recent compositional data analyses have linked extracurricular activity allocation with academic performance, daily allocations of sleep, physical activity, screen-related behavior, and learning with subjective well-being, and daily activity composition with problematic Internet use among university students (Chen et al., 2025; Wang et al., 2025; Zhang et al., 2023). This perspective extends behavioral-displacement accounts by situating digital behavior and exercise within the broader configuration of daily activities. The present study examines PSU and EA as behavioral tendencies rather than mutually exclusive time-use components and therefore does not constitute a compositional data analysis.

The reverse direction is also theoretically plausible. A stronger self-reported exercise-adherence tendency may be prospectively associated with lower subsequent PSU through greater daily structure, offline goal-directed activity, and fewer unstructured periods in which habitual smartphone checking can escalate. Exercise may also provide affective benefits and stress regulation (Rebar et al., 2015), thereby reducing reliance on smartphones for mood regulation, distraction, or social reassurance. Research on physical activity maintenance emphasizes self-regulatory processes, identity, affective experience, and routine formation (Rhodes and Sui, 2021). Recent work in student samples links persistence to exercise commitment, habit strength, autonomy support, and supportive physical-education contexts (Han et al., 2025; Huang and Jeong, 2025; Li et al., 2024; Rodrigues and Teixeira, 2023; Yan et al., 2025). Related synthesis work also suggests that mindfulness and self-regulatory capacities are inversely related to problematic smartphone use (Holtzman et al., 2025). EA should therefore be considered as a candidate behavioral routine that may precede lower subsequent PSU, while the causal mechanisms remain to be tested.

Existing empirical evidence generally supports a negative association between physical activity and problematic mobile phone or smartphone use, but important limitations remain. A systematic review and meta-analysis of adolescents and young adults found a significant negative association between physical activity and mobile phone addiction (Xiao et al., 2022). Most available studies, however, have relied on cross-sectional designs, making temporal direction difficult to establish. A recent three-wave cross-lagged study among Chinese college students found that physical activity was prospectively associated with lower later mobile phone addiction, whereas the reverse paths were not statistically significant (Wang et al., 2024). The traditional CLPM used in that study did not separate stable between-person differences from within-person change and focused on physical activity rather than self-reported exercise-adherence tendency. The present study extends this work by applying a model that separates between-person and within-person sources of association.

A central methodological problem in this literature is that many longitudinal models do not distinguish stable between-person differences from within-person temporal processes. For example, students who generally report high PSU may also generally report low EA because of stable individual characteristics, such as motivation, lifestyle, health status, or academic demands. This between-person association is meaningful, but it does not answer the more dynamic question of whether a student’s temporary increase in PSU is prospectively associated with a later change in that same student’s EA. Traditional cross-lagged panel models can confound these two levels of analysis, leading to estimates that may reflect stable differences between individuals rather than prospective changes within individuals (Hamaker et al., 2015). The random-intercept cross-lagged panel model (RI-CLPM) addresses this limitation by decomposing repeated measures into stable between-person components and time-specific within-person deviations (Hamaker et al., 2015; Hoffman and Hall, 2024; Mulder and Hamaker, 2021; Robitzsch and Lüdtke, 2025). In the present context, the model permits separate evaluation of stable between-person covariation and prospective associations between deviations from each participant’s own typical levels.

The present study used a four-wave longitudinal design to examine the reciprocal associations between PSU and EA among university students. By focusing on only two core constructs, the study aims to provide a parsimonious and theoretically focused test of the dynamic relationship between digital-behavior dysregulation and exercise maintenance. The four-wave design makes it possible to estimate autoregressive stability, cross-lagged paths, and time-invariant constraints across multiple adjacent intervals. Compared with cross-sectional mediation models or traditional CLPMs, this design is better suited to clarifying whether PSU and EA are linked primarily because of stable differences between students or because of prospective within-person dynamics.

Based on the theoretical and empirical considerations above, three hypotheses were specified. At the between-person level, the random intercept of PSU was expected to be negatively associated with the random intercept of EA. At the within-person level, higher-than-usual PSU was expected to be prospectively associated with lower subsequent EA, and higher-than-usual EA was expected to be prospectively associated with lower subsequent PSU. Whether the EA-to-PSU and PSU-to-EA cross-lagged associations differed in magnitude was examined as an exploratory question using time-specific within-person standardized coefficients and formal Wald tests (Schuurman et al., 2016).

**Figure 1:**
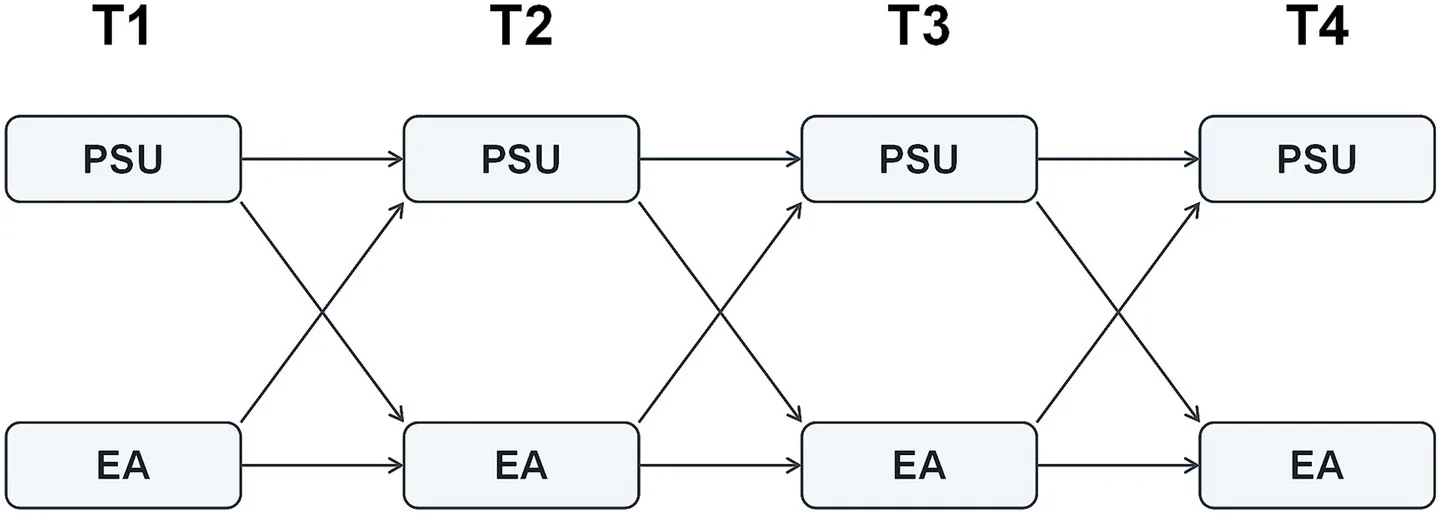
Hypothesized four-wave prospective associations between problematic smartphone use and exercise adherence. PSU, problematic smartphone use; EA, exercise adherence. Horizontal arrows represent hypothesized autoregressive associations, and diagonal arrows represent hypothesized cross-lagged associations. The arrows denote prospective associations to be tested and do not imply causal effects.

Accordingly, the conceptual reciprocal model treated PSU and EA as potentially linked through behavioral displacement, fragmented attention, structured routines, offline engagement, and self-regulatory organization (Figure 1). The arrows represent prospective associations to be tested rather than causal effects.

## 2 Materials and methods

### 2.1 Participants and procedure

This study used a four-wave longitudinal survey design to examine within-person reciprocal associations between PSU and EA among university students. Participants were recruited from six universities in Chengdu, Sichuan Province, China: Sichuan University, University of Electronic Science and Technology of China, Southwestern University of Finance and Economics, Southwest Jiaotong University, Chengdu Technological University, and Xihua University. Recruitment followed an open, multisite convenience approach. Campus contact persons and student associations assisted with survey access and distribution, and all students present at the survey settings who met the eligibility criteria were invited. No formal university-specific quotas were used.

Participant-level university identity and classroom, course, or survey-session identifiers were not retained in the de-identified analytic dataset to reduce disclosure risk. University type remained available as a broad classification but was not equivalent to specific site identity. No site identity was inferred from other variables. Consequently, site-specific sample sizes, university-level intraclass correlations, and university or classroom fixed-effect sensitivity analyses were not estimable.

The project began in March 2025. The baseline assessment was conducted in March 2025 (T1), and follow-up assessments were administered at approximately three-month intervals in June 2025 (T2), September 2025 (T3), and December 2025 (T4). The baseline-to-final observation period covered approximately 9 months. Participants reported experiences during the preceding 4 weeks, and adjacent reference windows did not overlap. The three-month spacing was chosen to align the temporal design with medium-term behavioral tendencies (Collins, 2006; Dormann and Griffin, 2015). The three intervals corresponded to March–June, June–September, and September–December; therefore, elapsed time, academic-calendar conditions, and season could not be separated within this single cohort.

Participants were eligible if they were full-time undergraduate students at one of the participating universities, were at least 18 years old, and provided written informed consent. The first page of the questionnaire clearly stated that the survey was open only to adults aged 18 years or above. Students who did not confirm that they were at least 18 years old or who did not provide written informed consent were not enrolled. All participants were informed that participation was voluntary, that they could withdraw at any time without penalty, and that their responses would be used only for academic research. No minors were included in the study.

The study protocol was reviewed and approved by the Ethics Committee of the Department of Physical Education, Chengdu Technological University, China (approval number: CDTU2025TY012). The approval and written informed-consent process covered longitudinal linkage and de-identified research use. Trained research assistants received standardized instructions regarding recruitment, consent, questionnaire administration, confidentiality, and data-quality screening. Paper-and-pencil questionnaires were administered in campus teaching or activity spaces under research-assistant supervision. Each wave remained open for 1 week. No monetary compensation, gifts, vouchers, or course credit were offered. The baseline questionnaire took approximately 10–15 min and each follow-up approximately 6–10 min.

At each wave, participants used the same fixed study-specific longitudinal code to link repeated questionnaires. The linkage file was stored separately from the analytic data and was accessible only to authorized research-team members. The analytic dataset contained no names, mobile-phone numbers, student numbers, participant-level university identifiers, or linkage codes. No cryptographic hashing or encryption procedure for the code was documented; confidentiality relied on separate storage, restricted access, and exclusion of linkage information from the analytic dataset. Because the broader longitudinal project remains ongoing, the linkage file is retained only for approved follow-up matching and will be destroyed after completion of the longitudinal project and final data verification.

Quality-control procedures were defined before the structural analyses. Baseline questionnaires were excluded for lack of written consent or age confirmation, completion of fewer than 80% of substantive items, insufficient item data to compute valid baseline PSU or EA scores, failure of the instructed-response item, patterned or careless responding, internally inconsistent or impossible responses, or duplicate, invalid, or unmatched longitudinal codes. The instructed-response and response-pattern checks followed recommended survey-quality practices (Meade and Craig, 2012). A total of 159 returned questionnaires met at least one exclusion criterion. Criterion-level screening was documented during data cleaning, but participant-level site identifiers were not retained; consequently, site-specific recruitment, exclusion, and follow-up counts could not be reconstructed.

For scale scoring, a wave-specific score was computed when at least 80% of the items in that scale were completed; the mean of available completed items was used, and the score was coded missing otherwise. Complete cases had valid PSU and EA scores at all four waves (*n* = 953). Wave-level nonparticipation was defined as absence of both focal scores. Follow-up questionnaires not returned by the end of the one-week collection window were treated as unavailable. Personal reasons for nonreturn were not systematically recorded. Participants were not excluded from longitudinal analyses solely because of incomplete follow-up.

At baseline, 1,500 eligible students were approached and received questionnaire packets; 1,400 returned questionnaires, yielding a return proportion of 93.3%. After the 159 exclusions, 1,241 valid baseline records were retained. The target of approximately 1,500 was a pragmatic multisite recruitment goal intended to provide a large cohort for four-wave RI-CLPM estimation and anticipated follow-up loss; it was not based on a prospectively registered power calculation. A post-data Monte Carlo design-sensitivity analysis is reported in Supplementary Table S13 and is not presented as an *a priori* power analysis.

### 2.2 Measurements

All measures were administered in Chinese. At each of the four waves, participants completed measures of problematic smartphone use (PSU) and exercise adherence (EA). To align the measures with the longitudinal design, participants were instructed to answer all repeated measures with reference to their experiences during the past 4 weeks. Scale scores were computed by averaging the relevant items, with higher scores indicating higher levels of the target construct. The same item wording and response format were used across all waves to support subsequent longitudinal measurement-invariance testing.

#### 2.2.1 Demographic and background variables

At baseline, participants reported their gender, age, grade, academic major, university type, household registration status, geographic region of origin, ethnicity, only-child status, monthly disposable allowance, parental education, perceived socioeconomic status (SES), residence type, relationship status, and part-time job status. They also reported height and weight for calculating body mass index (BMI), self-rated health, recent sports injury or exercise limitation, sport identity, organized exercise frequency, daily smartphone-use duration, regular exercise habit, and campus sports participation. The preferred covariate-adjusted RI-CLPM included baseline gender, age, subjective SES, household registration, ethnicity, BMI category, self-rated health, and injury status. For the RI-CLPM covariate analyses, categorical predictors were represented by dummy indicators using male, age 18 years, middle SES, urban registration, Han ethnicity, normal weight, good self-rated health, and no injury as reference categories. This dummy coding applied to the RI-CLPM sensitivity models; the separate missingness diagnostics used ordered trend scores for selected ordinal variables, as detailed in Supplementary Table S1. Additional sensitivity specifications incorporated academic major, university type, and region; daily smartphone-use duration, exercise habit, and organized exercise frequency; or allowed the core covariates to predict the T1 within-person deviation factors. Exact coding for the RI-CLPM covariates is reported in Supplementary Table S5.

#### 2.2.2 Problematic smartphone use

Problematic smartphone use was assessed using the Smartphone Addiction Scale—Chinese Short Version (SAS-CSV). The SAS-CSV was translated and validated by Zhao et al. (2022) based on the original Smartphone Addiction Scale—Short Version (SAS-SV) developed by Kwon et al. (2013). Although the scale title uses the term “addiction,” the present study treated the score as a continuous indicator of problematic smartphone use rather than as a clinical diagnosis. The original SAS-SV consists of 10 items selected from the longer Smartphone Addiction Scale and was developed as an efficient screening instrument for smartphone addiction risk (Kwon et al., 2013). The Chinese validation study supported a three-factor structure among Chinese college students: tolerance with four items, withdrawal with three items, and negative effect with three items; the total score can also be used as a global indicator of smartphone-addiction tendency (Zhao et al., 2022).

Participants rated each item on a 6-point Likert scale ranging from 1 = strongly disagree to 6 = strongly agree. A representative item is: “Using my smartphone longer than I had intended.” Higher mean scores indicate more severe problematic smartphone use. Previous evidence has shown good reliability and validity for the SAS-SV and SAS-CSV. Kwon et al. (2013) reported strong internal consistency for the SAS-SV, and Zhao et al. (2022) reported good internal consistency, test–retest reliability, structural validity, convergent validity, and discriminant validity for the SAS-CSV in Chinese college students. Total-scale mean scores were used in the primary observed-score RI-CLPM. For the measurement-invariance analyses and multiple-indicator latent RI-CLPM, the tolerance, withdrawal, and negative-effect subscale means served as three indicators at each wave.

#### 2.2.3 Exercise adherence

Exercise adherence was measured using the 14-item Exercise Adherence Scale (EAS) developed by Wang et al. (2016). In this study, the total score is interpreted as a self-reported exercise-adherence or exercise-maintenance tendency rather than objectively maintained physical activity. The scale contains three correlated dimensions: behavioral habit (4 items), effort input (5 items), and emotional experience (5 items), consistent with applications in general Chinese student samples (Tian and Shi, 2022).

Participants rated each item from 1 = strongly disagree to 5 = strongly agree with reference to the preceding 4 weeks. No separate not-applicable response option was present; all eligible participants were asked to respond, and low endorsement was represented by low item scores. A representative item is: “I try to eliminate distractions to keep exercising.” Higher means indicate a stronger self-reported exercise-adherence tendency. Total-scale means were used in the primary observed-score RI-CLPM; the three subscale means were used for longitudinal invariance and the multiple-indicator latent RI-CLPM. Distributional analyses by baseline exercise habit and an exerciser-only RI-CLPM were added as sensitivity analyses.

### 2.3 Data analysis

All structural equation models were estimated in Mplus version 8.3 (Muthén and Muthén, 1998–2017) using robust maximum likelihood (MLR), two-sided tests, and 95% confidence intervals. FIML used all available repeated PSU and EA outcomes under a missing-at-random working assumption (Enders and Bandalos, 2001; Newman, 2014). Preliminary scoring, descriptive statistics, reliability estimates, pairwise-complete correlations, missingness summaries, and multiple-imputation preparation were conducted with the cleaned analytic data. The study was not prospectively registered. An internal analysis plan developed before structural modeling specified the observed-score RI-CLPM and M1–M4 model set, although the plan was not publicly registered or independently time-stamped. Component-specific equality models, directional standardization analyses, missing-data sensitivity analyses, EAS subgroup analyses, and the design-sensitivity simulation were conducted after evaluation of the primary model set; the partial-equality model was *post hoc*.

Participant flow and construct-specific availability were summarized for every wave. Exploratory logistic regressions examined observed predictors of absence of both focal scores at any follow-up and at T4 (Supplementary Table S1). The primary RI-CLPM likelihood contained the eight repeated PSU and EA scores. Missing-data robustness was then evaluated with a simplified auxiliary-variable FIML model incorporating gender, age, subjective SES, BMI category, self-rated health, injury status, organized exercise frequency, daily smartphone-use duration, and exercise habit, and with delta-adjusted pattern-mixture multiple imputation (50 imputations, 20 iterations, predictive mean matching, fixed seed 20260723). Complete-case agreement was treated as limited reassurance; none of these analyses was interpreted as verification of MAR.

The auxiliary-variable model was specified as a supplementary missing-data sensitivity analysis rather than a replacement for the primary model. Because the simplified saturated-correlates specification retained a nonpositive-definite PSI warning, its estimates were interpreted together with the independent multiple-imputation delta analysis. The latter imposed joint shifts of up to one observed standard deviation on originally missing PSU and EA values, with imputed scores restricted to their theoretical ranges.

Means, standard deviations, Cronbach’s alpha, model-consistent omega total, and zero-order correlations were calculated for repeated PSU and EA scores. Omega total was derived from wave-specific correlated three-factor CFAs for the unit-weighted total score. A baseline comparison of the theoretical six-factor structure with a single-factor alternative was retained as a discriminant-structure check. Poor fit of the single-factor alternative was not interpreted as evidence that common method bias was absent.

Longitudinal measurement invariance was tested separately for PSU and EA using three subscale-mean indicators at each wave. Marker-variable scaling fixed the first loading at each wave. Metric models constrained corresponding loadings across waves; scalar models additionally constrained indicator intercepts, fixed the T1 latent mean to zero, and freely estimated T2–T4 latent means. Indicator residual variances were freely estimated, and all six same-subscale residual covariances across wave pairs were freely estimated. Configural, metric, and scalar models were evaluated using χ^2^, CFI, TLI, RMSEA with 90% confidence intervals, and SRMR (Hu and Bentler, 1999). Invariance decisions used established changes in CFI, RMSEA, and SRMR (Chen, 2007; Cheung and Rensvold, 2002). Scalar invariance supported longitudinal comparison at the subscale-indicator level (Meredith, 1993; Putnick and Bornstein, 2016; Vandenberg and Lance, 2000), although aggregation may conceal item-level noninvariance.

The primary longitudinal analysis used a two-variable, four-wave RI-CLPM based on observed total-scale mean scores. Separate random intercepts for PSU and EA had unit loadings on their four repeated measures. Single-indicator latent deviation factors represented time-specific within-person departures from each participant’s usual level, and the residual variances of the observed repeated scores were fixed to zero. This specification therefore treated the observed scale means as measured without error. The random intercepts were allowed to correlate, within-wave deviations were allowed to covary, and adjacent-wave autoregressive and cross-lagged paths were estimated (Hamaker et al., 2015; Mulder and Hamaker, 2021).

A conventional CLPM was estimated as a benchmark. The primary RI-CLPM model set comprised M1, with freely estimated autoregressive and cross-lagged paths; M2, with corresponding autoregressive paths constrained; M3, with corresponding cross-lagged paths constrained; and M4, with both sets constrained. Component-specific equality models were subsequently examined to identify which pathway sets could be constrained without a significant loss of fit. A partial-equality model combining the supported PSU-to-EA and EA-autoregressive constraints was then evaluated *post hoc* as a sensitivity specification. Model comparison considered absolute fit, AIC, BIC, robust scaled χ^2^ difference tests, parsimony, and substantive interpretability (Akaike, 1974; Satorra and Bentler, 2001; Schwarz, 1978).

Direct Mplus STDYX estimates were used to describe the primary paths. Directional differences were evaluated using time-specific within-person standardized coefficients. For each interval, the EA-to-PSU coefficient was calculated as bEA → PSU × √[Var(wEA_t)/Var(wPSU_t + 1)], and the reverse coefficient was standardized analogously. Paths, variances, covariances, and their uncertainty entered Mplus MODEL CONSTRAINT, and robust Wald tests used the delta method. Pooled within-person and pooled observed-total-score standardizations were retained as alternative scaling checks (Schuurman et al., 2016).

Sensitivity analyses included complete-case, post hoc partial-equality, multiple-indicator latent, theory-driven covariate-adjusted, exerciser-only, and none-versus-any-exercise multigroup RI-CLPMs. The preferred covariate model used gender, age, subjective SES, household registration, ethnicity, BMI category, self-rated health, and injury status as predictors of the random intercepts; broader specifications added academic/contextual and construct-proximal variables or T1 within-person paths. A post-data Monte Carlo design-sensitivity analysis calibrated to *N* = 1,241 and the aggregate empirical missing-pattern distribution evaluated detection, bias, standard-error bias, and 95% coverage; it was not an *a priori* or observed-power analysis. Participant-level university and classroom/session clustering could not be modeled because those identifiers were not retained.

## 3 Results

### 3.1 Participant flow, baseline characteristics, and missingness

**Figure 2:**
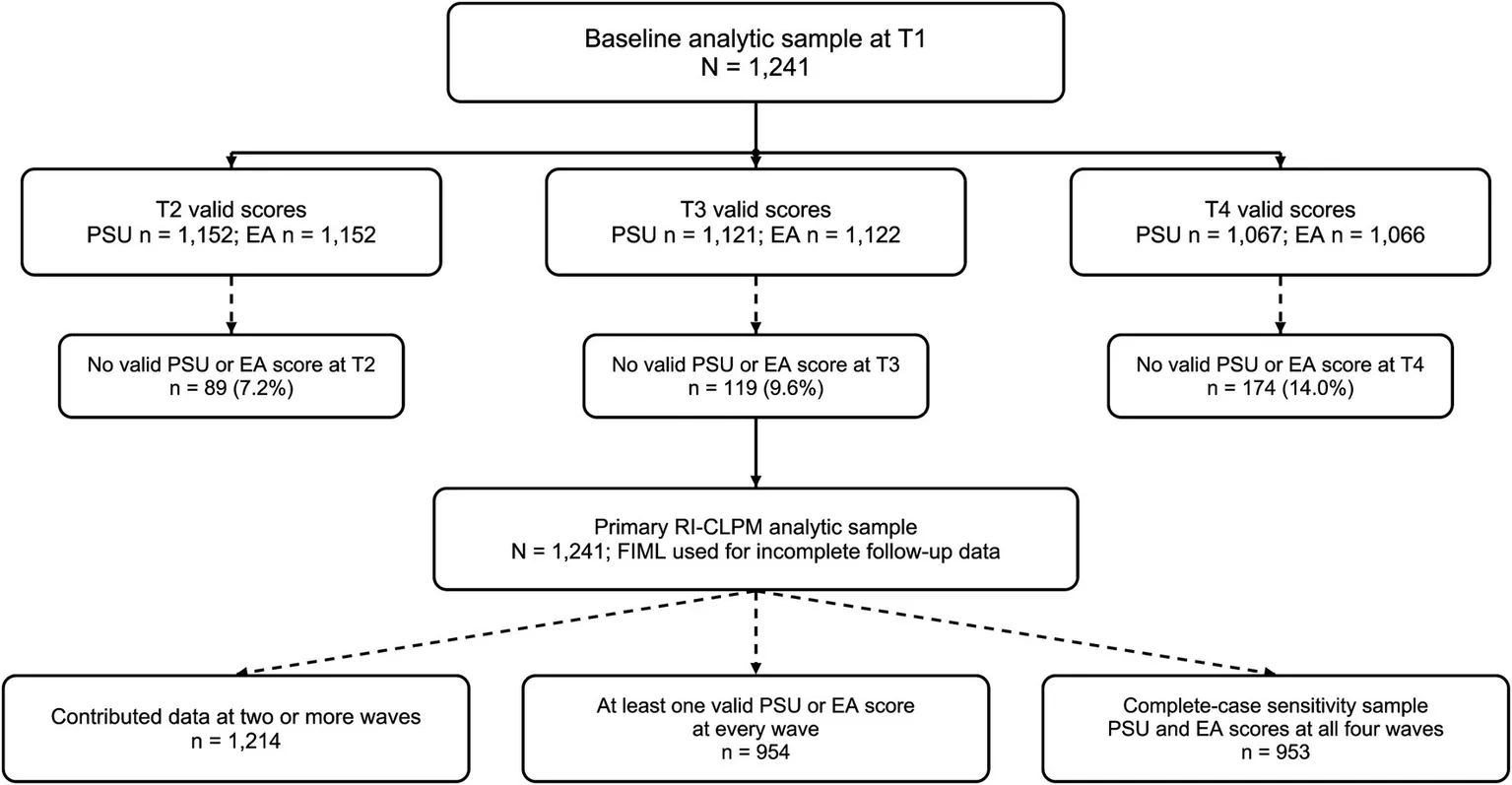
Participant flow and availability of problematic smartphone use and exercise adherence scores across four waves. PSU, problematic smartphone use; EA, exercise adherence; FIML, full-information maximum likelihood. Construct-specific valid-score counts are shown for each follow-up wave. The primary RI-CLPM retained all 1,241 participants with valid baseline data. “No valid PSU or EA score” denotes a wave at which neither focal score was available. The count of 954 therefore refers to participants with at least one valid focal score at every wave, whereas the complete-case sensitivity analysis required both PSU and EA scores at all four waves (*n* = 953).

The analytic baseline cohort comprised 1,241 participants. Valid PSU counts were 1,241, 1,152, 1,121, and 1,067 at T1–T4; corresponding EA counts were 1,241, 1,152, 1,122, and 1,066. A total of 953 participants had valid scores for both constructs at all four waves. Among 288 participants with at least one missing follow-up outcome, 287 lacked both constructs at one or more waves and one lacked EA only. Joint missingness was monotone for 169 participants and nonmonotone for 119. Follow-up nonreturn by the end of a collection window was recorded as unavailable, but personal reasons for nonreturn were not systematically collected (Figure 2; Supplementary Tables S1, S9, S10).

The cohort included students from different grades, academic disciplines, university types, socioeconomic backgrounds, and health and exercise profiles (Table 1). Extended smartphone use was common, whereas regular and stable exercise routines were less prevalent.

**Table 1:** Sample characteristics of the analytic baseline cohort.

Characteristic	Category	*n*	Percent
Gender	Male	573	46.2
	Female	662	53.3
	Other/prefer not to say	6	0.5
Age	18 years	177	14.3
	19 years	345	27.8
	20 years	339	27.3
	21 years	259	20.9
	22 years or above	121	9.8
Grade	Freshman	349	28.1
	Sophomore	348	28.0
	Junior	339	27.3
	Senior	205	16.5
Major	STEM	399	32.2
	Humanities/social sciences/education	305	24.6
	Economics/management	272	21.9
	Medicine/health	116	9.3
	Arts	59	4.8
	Physical education	33	2.7
	Other/prefer not to say	57	4.6
University type	Comprehensive university	487	39.2
	STEM-oriented university	338	27.2
	Applied university	177	14.3
	Specialized university	239	19.3
Household registration	Urban	722	58.2
	Rural	519	41.8
Region	Eastern China	484	39.0
	Central China	371	29.9
	Western China	310	25.0
	Northeastern China	76	6.1
Monthly allowance	≤1,000 yuan	85	6.8
	1,001–1,500 yuan	336	27.1
	1,501–2,000 yuan	464	37.4
	2,001–3,000 yuan	272	21.9
	≥3,001 yuan	84	6.8
Parental education	Junior high school or below	297	23.9
	Senior high/technical secondary	368	29.7
	Junior college	259	20.9
	Bachelor’s degree	258	20.8
	Master’s degree or above	59	4.8
Subjective SES	Low	290	23.4
	Middle	654	52.7
	High	297	23.9
BMI category	Underweight	199	16.0
	Normal weight	825	66.5
	Overweight	176	14.2
	Obesity	41	3.3
Self-rated health	Poor	56	4.5
	Fair	334	26.9
	Good	642	51.7
	Very good	209	16.8
Sports identity	Non-sports student	1,127	90.8
	Sports club member	81	6.5
	PE major/school team	33	2.7
Organized exercise frequency	None	270	21.8
	Once per week	555	44.7
	Twice per week	282	22.7
	Three or more times per week	134	10.8
Daily smartphone use	2 h or less	49	3.9
	2–4 h	301	24.3
	4–6 h	472	38.0
	6–8 h	287	23.1
	More than 8 h	132	10.6
Exercise habit	None	457	36.8
	Occasional exercise	452	36.4
	Relatively stable habit	288	23.2
	Regular training	44	3.5

Percentages are based on the analytic baseline cohort, *N* = 1,241. Percentages may not sum exactly to 100 because of rounding. SES, socioeconomic status; BMI, body mass index; STEM, science, technology, engineering, and mathematics; PE, physical education.

Exploratory logistic models identified several nominal baseline correlates of wave-level nonparticipation (Supplementary Table S1), but individual *p* values were not multiplicity-adjusted and were not treated as confirmatory risk-factor findings. The primary FIML likelihood used the eight repeated PSU and EA scores. The auxiliary-variable FIML and delta-adjustment analyses are reported with the sensitivity results in Section 3.7. The missingness pattern was inconsistent with simple MCAR, while MAR remained an unverifiable working assumption.

### 3.2 Descriptive statistics, reliability, and baseline discriminant-structure check

The sample means of PSU and EA changed only modestly across waves (Table 2). Cronbach’s alpha ranged from 0.869 to 0.876 for PSU and from 0.875 to 0.885 for EA. Model-consistent omega total from the correlated three-factor models ranged from 0.895 to 0.901 for PSU and from 0.897 to 0.905 for EA. Repeated scores of the same construct were moderately correlated, and most zero-order PSU–EA associations were negative. These descriptive correlations combine between-person and within-person variation and were not interpreted as prospective within-person effects.

**Table 2:** Descriptive statistics, internal consistency, and zero-order correlations for problematic smartphone use and exercise adherence.

No.	Variable	*n*	M	SD	α	ω	1	2	3	4	5	6	7	8
1	PSU_T1	1,241	3.434	1.043	0.875	0.901	—							
2	PSU_T2	1,152	3.453	1.012	0.869	0.895	0.508	—						
3	PSU_T3	1,121	3.517	1.034	0.874	0.899	0.408	0.521	—					
4	PSU_T4	1,067	3.542	1.045	0.876	0.901	0.352	0.445	0.600	—				
5	EA_T1	1,241	3.431	0.777	0.879	0.900	−0.198	−0.292	−0.159	−0.140	—			
6	EA_T2	1,152	3.471	0.764	0.875	0.898	−0.090	−0.221	−0.230	−0.190	0.460	—		
7	EA_T3	1,122	3.409	0.796	0.885	0.905	−0.066	−0.097	−0.203	−0.225	0.331	0.497	—	
8	EA_T4	1,066	3.447	0.782	0.878	0.897	−0.011	−0.065	−0.134	−0.191	0.300	0.369	0.492	—

PSU, problematic smartphone use; EA, exercise adherence; α, Cronbach’s alpha; ω, model-consistent omega total for the unit-weighted total score derived from the correlated three-factor model. Correlations are shown below the diagonal and were calculated using pairwise-complete observations; pairwise sample sizes ranged from 1,007 to 1,241. The n column gives the number with a valid scale mean at each wave. Alpha was based on pairwise-complete item covariance matrices; omega used the wave-specific correlated three-factor CFA. Model fit and confidence intervals for omega are reported in Supplementary Table S11.

The baseline theoretical six-factor structure provided a substantially better representation of the 24-item covariance matrix than the single-factor alternative (Supplementary Table S2). This comparison indicates that a single general factor was insufficient to represent the item structure; it does not rule out shared response tendencies or longitudinal common method variance.

### 3.3 Longitudinal measurement invariance

Longitudinal invariance used tolerance, withdrawal, and negative effect as PSU indicators and behavioral habit, effort input, and emotional experience as EA indicators. Configural, metric, and scalar models retained acceptable or excellent fit (Table 3). Scalar-model fit was χ^2^(42) = 52.276, *p* = 0.133, CFI = 0.998, TLI = 0.996, RMSEA = 0.014, 90% CI [0.000, 0.025], SRMR = 0.023 for EA and χ^2^(42) = 60.205, *p* = 0.034, CFI = 0.996, TLI = 0.994, RMSEA = 0.019, 90% CI [0.005, 0.029], SRMR = 0.027 for PSU. Scalar invariance was supported at the subscale-indicator level, while item-level invariance was not tested.

**Table 3:** Longitudinal measurement invariance based on subscale-mean indicators across four waves.

Construct	Model	χ^2^	df	*p*	CFI	TLI	RMSEA	RMSEA 90% CI	SRMR	ΔCFI	ΔRMSEA	ΔSRMR
EA	Configural	30.489	30	0.441	1.000	1.000	0.004	[0.000, 0.022]	0.013	—	—	—
EA	Metric	35.905	36	0.473	1.000	1.000	0.000	[0.000, 0.020]	0.018	0.000	−0.004	0.005
EA	Scalar	52.276	42	0.133	0.998	0.996	0.014	[0.000, 0.025]	0.023	−0.002	0.014	0.005
PSU	Configural	25.496	30	0.701	1.000	1.002	0.000	[0.000, 0.017]	0.011	—	—	—
PSU	Metric	41.761	36	0.235	0.999	0.998	0.011	[0.000, 0.024]	0.025	−0.001	0.011	0.014
PSU	Scalar	60.205	42	0.034	0.996	0.994	0.019	[0.005, 0.029]	0.027	−0.003	0.008	0.002

PSU, problematic smartphone use; EA, exercise adherence. PSU indicators were tolerance, withdrawal, and negative-effect subscale means; EA indicators were behavioral habit, effort input, and emotional experience. Models used MLR and marker-variable scaling. Metric models constrained corresponding loadings; scalar models also constrained indicator intercepts, fixed the T1 latent mean to zero, and freely estimated T2–T4 latent means. Residual variances were freely estimated. For each subscale indicator, residual covariances across all six pairs of waves were freely estimated. Change indices compare each model with the preceding model. The conclusion concerns subscale indicators and may not detect item-level noninvariance.

### 3.4 Model comparison and selection

The conventional CLPM fit substantially worse than the RI-CLPM specifications (Table 4). Evidence among the initial RI-CLPMs was mixed: M1 had the best absolute fit and lowest AIC, whereas BIC favored M4. Robust nested tests showed that the constraints in M2, M3, and M4 significantly reduced fit relative to M1.

**Table 4:** Comparison of the conventional cross-lagged panel model and observed-score RI-CLPM specifications.

Model	Constraints	χ^2^ (df)	*p*	CFI	TLI	RMSEA [90% CI]	SRMR	AIC	BIC	Scaled Δχ^2^ (df)	*p*
CLPM	Conventional CLPM	174.171 (12)	<0.001	0.928	0.838	0.104 [0.091, 0.118]	0.055	21867.67	22031.63	—	—
M1	All paths freely estimated	12.320 (9)	0.196	0.999	0.996	0.017 [0.000, 0.039]	0.016	21708.25	21887.58	Reference	—
M2	Autoregressive paths equal	34.050 (13)	0.001	0.991	0.980	0.036 [0.022, 0.051]	0.033	21720.51	21879.35	23.453 (4)	<0.001
M3	Cross-lagged paths equal	24.415 (13)	0.028	0.995	0.989	0.027 [0.009, 0.043]	0.022	21712.34	21871.17	12.073 (4)	0.017
M4	Autoregressive and cross-lagged paths equal	42.782 (17)	<0.001	0.989	0.982	0.035 [0.022, 0.048]	0.030	21721.25	21859.59	31.206 (8)	<0.001

CLPM, conventional cross-lagged panel model; RI-CLPM, random-intercept cross-lagged panel model; AIC, Akaike information criterion; BIC, Bayesian information criterion. All models were estimated using robust maximum likelihood. Robust scaled chi-square difference tests compare each nested RI-CLPM with M1. The conventional CLPM is not nested within M1 and was included as a benchmark. Lower AIC and BIC values indicate better relative fit. Component-specific equality tests and the *post hoc* partial-equality model are reported in Supplementary Table S6.

Component-specific tests located temporal heterogeneity in the EA-to-PSU and PSU-autoregressive paths (Supplementary Table S6). Equality constraints on PSU-to-EA and EA-autoregressive paths did not significantly reduce fit. The *post hoc* partial-equality model combined those constraints, did not differ significantly from M1, and had lower AIC and BIC. M1 remained the primary interval-specific model because it belonged to the initial analysis and did not depend on the *post hoc* component tests; M9 was retained as a parsimonious sensitivity model.

### 3.5 Primary observed-score RI-CLPM findings

**Figure 3:**
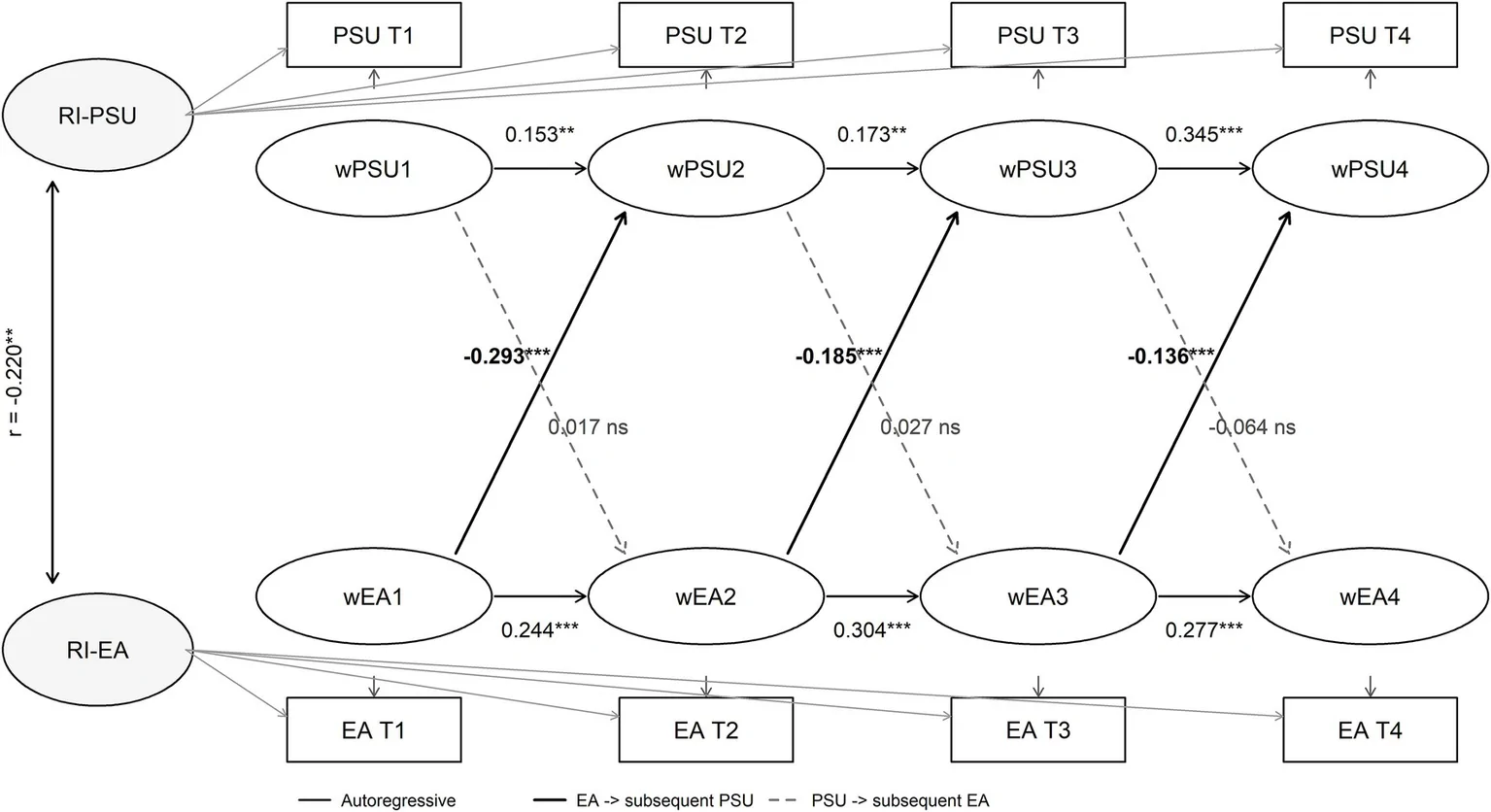
Standardized estimates from the primary observed-score random-intercept cross-lagged panel model. Values are direct Mplus STDYX estimates. Solid arrows represent statistically significant paths and dashed arrows nonsignificant paths. *RI_PSU* and *RI_EA* are random intercepts; *wPSU* and *wEA* are within-person latent deviations. Fixed unit loadings are omitted. Within-wave covariances are omitted for readability and reported in Supplementary Table S12. Paths are prospective associations and do not imply causal effects. **p < 0.01, ***p < 0.001.

The primary observed-score RI-CLPM is presented in Figure 3, with focal structural estimates in Table 5. The random intercepts were negatively correlated in this model, indicating that participants with higher stable PSU scores tended to report lower stable EA scores across the study period. Within-person deviations in both constructs also showed significant carryover across adjacent waves, with the strongest PSU autoregressive association occurring during the final interval.

**Table 5:** Unstandardized and standardized estimates from the primary observed-score RI-CLPM.

Path	Interval	*b*	SEb	β/r	SEstd	95% CI for β/r	pstd
RI_PSU ↔ RI_EA	Between-person	−0.057	0.019	−0.220	0.065	[−0.348, −0.092]	0.001
wPSU1 → wPSU2	T1–T2	0.144	0.043	0.153	0.045	[0.065, 0.240]	0.001
wPSU2 → wPSU3	T2–T3	0.180	0.052	0.173	0.050	[0.075, 0.271]	0.001
wPSU3 → wPSU4	T3–T4	0.355	0.040	0.345	0.039	[0.267, 0.422]	<0.001
wEA1 → wEA2	T1–T2	0.238	0.044	0.244	0.044	[0.158, 0.329]	<0.001
wEA2 → wEA3	T2–T3	0.321	0.047	0.304	0.044	[0.217, 0.391]	<0.001
wEA3 → wEA4	T3–T4	0.271	0.039	0.277	0.040	[0.199, 0.356]	<0.001
wPSU1 → wEA2	T1–T2	0.013	0.033	0.017	0.043	[−0.067, 0.102]	0.687
wPSU2 → wEA3	T2–T3	0.023	0.037	0.027	0.043	[−0.059, 0.112]	0.540
wPSU3 → wEA4	T3–T4	−0.052	0.032	−0.064	0.040	[−0.142, 0.014]	0.106
wEA1 → wPSU2	T1–T2	−0.350	0.049	−0.293	0.041	[−0.373, −0.213]	<0.001
wEA2 → wPSU3	T2–T3	−0.235	0.056	−0.185	0.044	[−0.271, −0.099]	<0.001
wEA3 → wPSU4	T3–T4	−0.169	0.046	−0.136	0.037	[−0.208, −0.064]	<0.001

PSU, problematic smartphone use; EA, exercise adherence; RI, random intercept; w, within-person latent deviation. b and SEb are unstandardized estimates and standard errors. β denotes a direct Mplus STDYX regression coefficient, and r denotes the direct STDYX random-intercept correlation. SEstd, confidence intervals, and pstd correspond to the direct STDYX estimates.

The cross-lagged results showed a consistent directional pattern. Higher-than-usual EA was prospectively associated with lower-than-usual PSU at every subsequent wave, whereas the PSU-to-EA estimates were small and all associated confidence intervals included zero. The EA-to-PSU point estimates became less negative over time, a pattern examined formally in the next section.

### 3.6 Directional and temporal comparisons

Time-specific within-person standardized comparisons showed significant directional differences at T1–T2 (difference = −0.310, SE = 0.054, 95% CI [−0.417, −0.204], *p* < 0.001) and T2–T3 (difference = −0.212, SE = 0.057, 95% CI [−0.323, −0.100], *p* < 0.001), but not T3–T4 (difference = −0.072, SE = 0.051, 95% CI [−0.173, 0.029], *p* = 0.161). The joint directional test was significant, Wald χ^2^(3) = 59.159, *p* < 0.001 (Table 6). Pooled within-person and pooled observed-SD standardizations produced the same interval-specific interpretation (Supplementary Table S7).

**Table 6:** Time-specific within-person-standardized Wald tests of directional differences and temporal variation.

Panel A: Directional differences within each interval					
Interval	Comparison	Difference	SE	95% CI	*p*
T1–T2	EA1 → PSU2 minus PSU1 → EA2	−0.310	0.054	[−0.417, −0.204]	<0.001
T2–T3	EA2 → PSU3 minus PSU2 → EA3	−0.212	0.057	[−0.323, −0.100]	<0.001
T3–T4	EA3 → PSU4 minus PSU3 → EA4	−0.072	0.051	[−0.173, 0.029]	0.161

Panel B: Temporal comparisons of the EA-to-PSU associations				
Comparison	Difference	SE	95% CI	*p*
T1–T2 minus T2–T3	−0.108	0.053	[−0.212, −0.004]	0.041
T1–T2 minus T3–T4	−0.157	0.053	[−0.260, −0.054]	0.003
T2–T3 minus T3–T4	−0.049	0.053	[−0.154, 0.056]	0.361

Coefficients were standardized at the within-person level using time-specific model-implied variances. For each interval, the regression coefficient was multiplied by the ratio of the predictor within-person standard deviation to the subsequent outcome within-person standard deviation. Paths, variances, covariances, and scaling uncertainty entered Mplus MODEL CONSTRAINT, and standard errors and confidence intervals used the delta method. Negative differences indicate that the EA-to-PSU association was more negative than the reverse association. The joint directional Wald test was χ^2^(3) = 59.159, *p* < 0.001; the joint test of equality among the three EA-to-PSU coefficients was χ^2^(2) = 9.378, *p* = 0.009. Alternative pooled within-person and pooled observed-SD comparisons are reported in Supplementary Table S7.

The EA-to-PSU associations differed across intervals, joint Wald χ^2^(2) = 9.378, *p* = 0.009. T1–T2 was more negative than T2–T3 (difference = −0.108, 95% CI [−0.212, −0.004], *p* = 0.041) and T3–T4 (difference = −0.157, 95% CI [−0.260, −0.054], *p* = 0.003), whereas T2–T3 and T3–T4 did not differ (difference = −0.049, 95% CI [−0.154, 0.056], *p* = 0.361).

### 3.7 Sensitivity analyses

The complete-case model (*n* = 953) and the post hoc partial-equality model reproduced the qualitative six-path pattern (Supplementary Table S3). Complete-case agreement was interpreted as limited reassurance rather than evidence that MAR held. The multiple-indicator latent RI-CLPM also retained three negative EA-to-PSU paths and three nonsignificant PSU-to-EA paths after modeling measurement error; its random-intercept association was attenuated and nonsignificant.

**Figure 4:**
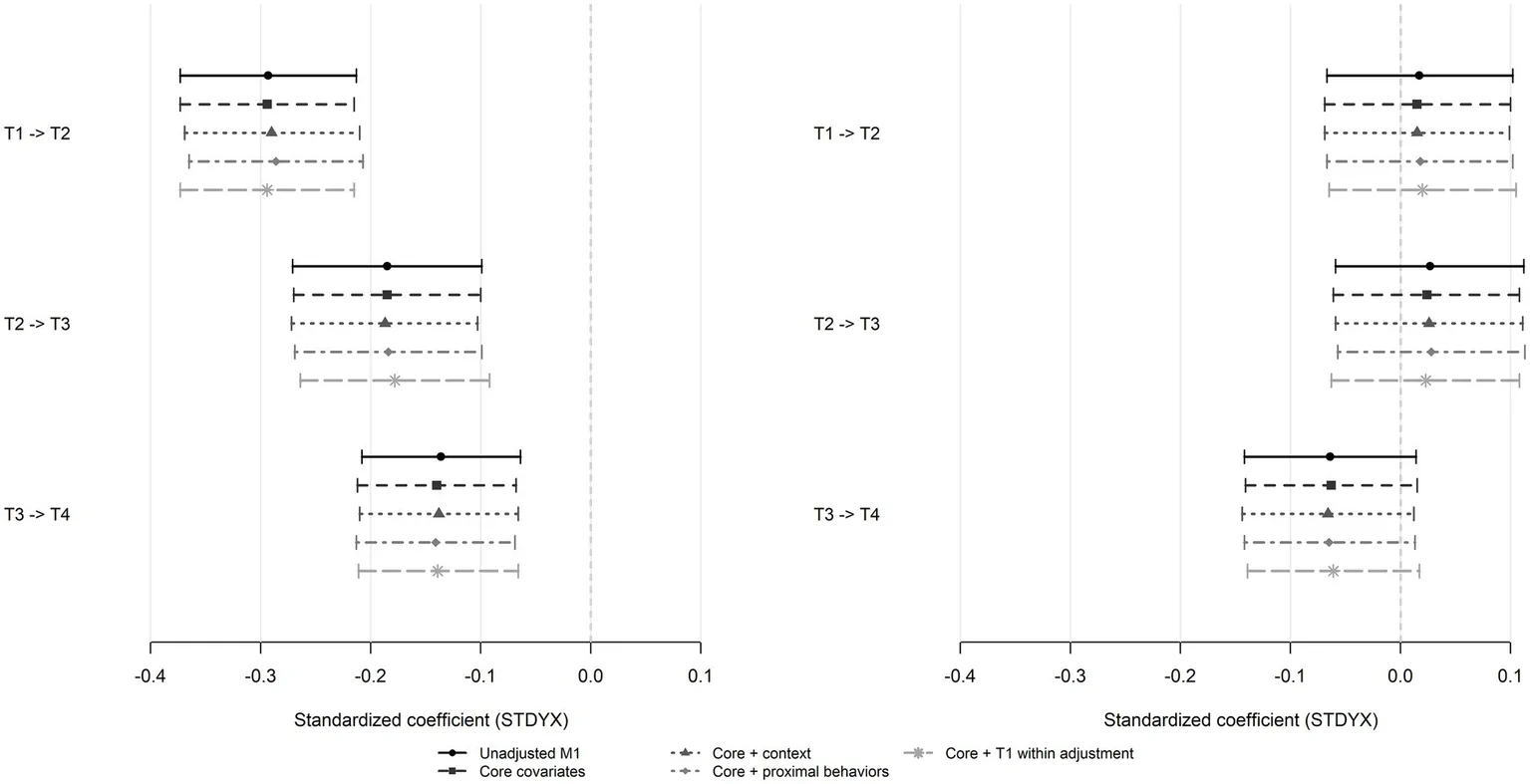
Standardized cross-lagged associations across alternative covariate specifications: (A) EA to subsequent PSU and (B) PSU to subsequent EA. Points are direct Mplus STDYX estimates and horizontal bars are two-sided 95% confidence intervals. The core model adjusted the PSU and EA random intercepts for baseline gender, age, subjective socioeconomic status, household registration, ethnicity, BMI category, self-rated health, and injury status. The context-expanded model additionally included academic major, university type, and geographic region. The proximal-behavior model additionally included daily smartphone-use duration, exercise habit, and organized exercise frequency. The alternate-placement model also allowed core covariates to predict the T1 within-person deviation factors. Point symbols and line patterns preserve interpretability in grayscale. EA, exercise adherence; PSU, problematic smartphone use; RI-CLPM, random-intercept cross-lagged panel model.

The theory-driven core covariate model and broader contextual, construct-proximal, and alternate-placement specifications produced only minimal cross-lagged changes (Figure 4; Tables 7; Supplementary Tables S4–S5). Across these models, all EA-to-PSU paths remained negative and significant, and all PSU-to-EA paths remained nonsignificant.

**Table 7:** Focal standardized estimates from the preferred core covariate-adjusted RI-CLPM.

Path	Interval	β/r	SE	95% CI	*p*
EA1 → PSU2	T1–T2	−0.294	0.040	[−0.373, −0.215]	<0.001
EA2 → PSU3	T2–T3	−0.185	0.043	[−0.270, −0.100]	<0.001
EA3 → PSU4	T3–T4	−0.140	0.037	[−0.212, −0.068]	<0.001
PSU1 → EA2	T1–T2	0.015	0.043	[−0.069, 0.100]	0.724
PSU2 → EA3	T2–T3	0.024	0.043	[−0.061, 0.108]	0.587
PSU3 → EA4	T3–T4	−0.063	0.040	[−0.141, 0.015]	0.114
RI_PSU ↔ RI_EA	Between-person	−0.210	0.069	[−0.345, −0.075]	0.002

PSU, problematic smartphone use; EA, exercise adherence; RI, random intercept. Values are direct Mplus STDYX estimates from the preferred core covariate-adjusted model. Both random intercepts were regressed on baseline gender, age, subjective socioeconomic status, household registration, ethnicity, BMI category, self-rated health, and injury status. Model fit was *χ*^2^ (117) = 86.207, *p* = 0.985, CFI = 1.000, TLI = 1.000, RMSEA = 0.000, 90% CI [0.000, 0.000], and SRMR = 0.016. The raw Mplus TLI was 1.019 and was capped at 1.000 for display. Alternative covariate specifications are summarized in Supplementary Table S4 and Figure 4.

Sensitivity analyses addressing the interpretation of EA are reported in Supplementary Table S8. The no-exercise baseline group showed no exact floor at T1 (M = 3.438, SD = 0.778, median = 3.500; near-floor = 1.09%). After excluding 457 participants reporting no baseline exercise habit, the exerciser-only model (*n* = 784) retained negative EA-to-PSU paths (*β* = −0.307, −0.151, and −0.156; *p* ≤ 0.008) and nonsignificant PSU-to-EA paths (*β* = 0.003, 0.006, and −0.086; *p* ≥ 0.117); the maximum absolute change from the full sample was 0.034. None-versus-any-exercise multigroup tests did not detect cross-lagged differences, robust Δχ^2^(6) = 4.059, *p* = 0.669.

The simplified auxiliary-variable FIML model closely reproduced the primary cross-lagged estimates (maximum |Δβ| = 0.002; Supplementary Table S9). It terminated normally with positive latent variances and no correlations exceeding one, but retained a nonpositive-definite PSI warning and was therefore interpreted as a qualified sensitivity result.

Across delta-adjusted multiple-imputation scenarios, all three EA-to-PSU paths remained negative and significant through the joint adverse −1 SD/−1 SD scenario; the weakest path at that boundary was EA T3 → PSU T4, b = −0.107, 95% CI [−0.199, −0.016], *p* = 0.021. No tipping point was found through one standard deviation (Supplementary Table S10). These scenarios evaluate specified departures from MAR and do not identify the true missingness mechanism.

The post-data design-sensitivity simulation indicated that, under constant EA-to-PSU effects of |β| = 0.10 with null reverse paths, individual-path detection ranged from 0.634 to 0.783 and rejection of the joint directional Wald test was 0.955; all formal scenarios converged (Supplementary Table S13). This simulation describes the completed design and is not an *a priori* power calculation.

## 4 Discussion

### 4.1 Overview of the main findings

Across the primary model and the full set of sensitivity analyses, the same broad pattern emerged: within-person elevations in self-reported exercise-adherence tendency were prospectively associated with lower subsequent PSU, whereas within-person elevations in PSU were not reliably associated with subsequent EA. Formal directional differences were concentrated in the first two intervals and were not statistically significant from T3 to T4.

The pattern was reproduced under time-specific and pooled within-person standardization, pooled observed-score scaling, complete-case and auxiliary-variable FIML analyses, delta-adjusted missing-data scenarios, the multiple-indicator latent model, covariate adjustment, and the exerciser-only model. The negative random-intercept association in scale-score models was not reproduced in the multiple-indicator latent model, so the most defensible contribution remains the within-person finding rather than a definitive between-person conclusion.

### 4.2 Model-dependent between-person association between PSU and EA

The observed-score models indicated that students with higher stable PSU tended to report lower stable EA across the study period. This pattern is compatible with the possibility that digital dysregulation and weak exercise maintenance cluster within broader lifestyle profiles involving daily structure, time management, health orientation, sleep, academic demands, or self-regulatory routines. The covariate-adjusted observed-score model retained the negative residual association after accounting for measured baseline characteristics.

The association was substantially attenuated and nonsignificant when the two constructs were represented by multiple subscale indicators and measurement error was estimated explicitly. This difference cautions against treating the random-intercept correlation as a robust substantive finding. Between-person associations can depend on how stable trait variance and measurement error are represented, and they should not be interpreted as evidence that PSU causes poor EA or that EA causes lower PSU. The RI-CLPM remains valuable because it separates this model-dependent between-person covariance from the more consistent within-person prospective pattern (Berry and Willoughby, 2017; Curran and Bauer, 2011).

### 4.3 Prospective associations from self-reported exercise-adherence tendency to later problematic smartphone use

The repeated negative EA-to-PSU paths indicate that, when a participant reported a stronger exercise-adherence tendency than expected from their own stable level, they tended to report lower PSU at the next assessment. The association weakened over time: T1–T2 was more negative than T2–T3 and T3–T4, whereas the latter two intervals did not differ. The same direction was observed after excluding participants who reported no baseline exercise habit. These findings concern a self-reported tendency and do not demonstrate changes in objectively maintained physical activity.

Greater daily structure, offline goal-directed engagement, affect regulation, and self-regulatory organization are plausible interpretations of the EA-to-PSU association. Habit research suggests that behaviors repeated in stable contexts may become less dependent on momentary motivation (Lally et al., 2010; Wood and Rünger, 2016), and exercise-specific work identifies habit formation as an important component of maintenance (Kaushal and Rhodes, 2015). Motivational perspectives also link autonomous exercise motivation with persistence (Teixeira et al., 2012). These mechanisms were not measured as mediators, and the RI-CLPM does not establish that increasing EA would causally reduce PSU.

### 4.4 Interpreting the nonsignificant PSU-to-EA paths

The PSU-to-EA estimates provided limited evidence for a three-month prospective association after stable between-person differences were separated. Their confidence intervals included both small negative and small positive values, so the estimates should not be interpreted as proof that a PSU-to-EA process is absent.

Smartphone-related disruption may operate over shorter time scales than the present assessments captured. A period of dysregulated use may delay an individual workout, displace sleep, or increase sedentary leisure without producing a detectable three-month change in self-reported exercise-maintenance tendency. A global PSU score also cannot distinguish gaming, short-video viewing, social networking, academic use, and other activities that may have different relationships with exercise-related behavior. Daily diary, ecological momentary assessment, and device-based studies are needed to examine these shorter and activity-specific processes.

The nonsignificant paths also caution against a moralized account in which problematic smartphone use is assumed to make students abandon exercise. The supported conclusion is narrower: EA deviations were prospectively associated with subsequent PSU deviations at the assessed intervals, whereas the reverse prospective association was not detected.

### 4.5 Theoretical implications

The findings are consistent with a routine-based interpretation of the PSU–EA relationship. The conceptual model included a displacement pathway from PSU to lower EA and a routine-related pathway from EA to lower PSU. The observed within-person pattern was more consistent with the latter at the assessed lags, while leaving open the possibility that displacement occurs over shorter intervals or for specific forms of smartphone activity.

The results also demonstrate why level-specific, measurement-sensitive, and scale-sensitive interpretation matters. The conventional CLPM fit less well than the RI-CLPMs, and the random-intercept association was measurement dependent. The directional comparison remained similar under three defensible standardizations, but the interval-specific difference was not significant at T3–T4. Cross-sectional or traditional cross-lagged results could obscure whether an association reflects stable differences between students or change relative to an individual’s own level.

A compositional perspective offers a complementary direction for future work. RI-CLPM separates between-person and within-person processes, whereas compositional data analysis addresses allocation of a finite daily time budget. Combining repeated RI-CLPM assessments with 24-h time-use compositions could clarify whether changes in self-reported exercise-adherence tendency are accompanied by reallocations from screen-based leisure, sleep, study, or other activities (Chen et al., 2025; Wang et al., 2025; Zhang et al., 2023).

### 4.6 Practical implications

The results provide a rationale for evaluating support for exercise-related routine building as one component of university digital-well-being programs. Smartphones are embedded in academic communication, social connection, and daily organization, which can make restriction-only approaches difficult to sustain. The present model does not estimate the intervention effect of increasing EA on PSU, so practical proposals require randomized or quasi-experimental testing and objective activity measurement.

Campus programs could test stable exercise schedules, low-threshold group activities, peer support, and implementation-intention strategies that help students exercise at consistent times and places. Meta-analytic evidence indicates that university physical-activity interventions can improve activity levels, although maintenance remains challenging (Favieri et al., 2023; Yuan et al., 2024), and implementation intentions can support physical activity when plans include concrete cues and barrier-management strategies (Bélanger-Gravel et al., 2013; Peng et al., 2023).

Digital-well-being trials could examine planned phone-free exercise periods, exercise self-monitoring, and reflection on smartphone-use contexts. Such programs should monitor feasibility and unintended burden and should not present exercise adherence as an established treatment or preventive factor for PSU on the basis of the present observational findings.

## 5 Limitations and future directions

Several limitations qualify the findings. Both constructs were measured by self-report, creating potential recall error, social-desirability effects, and shared response tendencies (Podsakoff et al., 2003). The baseline six-factor versus single-factor comparison showed that a single general factor was inadequate, but it cannot rule out common method bias. Shared response tendencies could inflate cross-construct covariance, especially stable between-person covariance, whereas classical measurement error could attenuate lagged associations; the net direction of measurement bias is therefore uncertain. Objective smartphone logs, accelerometry, wearable data, and exercise-attendance records would strengthen future work (Ejaz et al., 2023; Hitcham et al., 2023; Parry et al., 2021; Prince et al., 2008; Shi et al., 2025).

EA was operationalized as a self-reported adherence or maintenance tendency rather than objectively sustained physical activity. Similar score distributions across baseline exercise-habit groups and the exerciser-only sensitivity analysis reduce concern that the main pattern was driven solely by participants reporting no exercise, but they do not establish content validity among non-exercisers. The primary RI-CLPM also treated observed scale means as measured without error. The multiple-indicator latent model reproduced the cross-lagged pattern but not the negative random-intercept association. In addition, scalar invariance was tested with subscale means and may have concealed item-level noninvariance.

FIML relies on a MAR working assumption that cannot be verified from observed data. Complete-case agreement provides limited reassurance and may itself be affected by selection. The auxiliary-variable model retained a PSI warning, and the delta-adjustment scenarios represent specified departures rather than identification of the true missingness mechanism. Participants who remained in follow-up may differ from those who did not on unmeasured characteristics; depending on how those characteristics relate to EA and PSU, selection could attenuate or exaggerate the observed paths, so its net direction cannot be determined.

Participant-level university identity and classroom/session identifiers were not retained. University type captured broad institutional context but could not quantify within-site dependence. If participants recruited within the same settings had positively correlated outcomes, model-based standard errors may be too small. This limitation also restricts site-specific generalizability. Future multisite studies should retain non-identifying site and classroom codes and account for clustering prospectively.

The study remains observational. RI-CLPM separates stable between-person differences from within-person deviations but does not eliminate time-varying confounding. Academic stress, sleep quality, depressive symptoms, social activities, changing injury status, weather, examinations, vacations, and campus schedules may affect both EA and PSU. The March, June, September, and December waves perfectly confounded elapsed time with season and academic calendar, so the observed interval differences cannot be attributed to a single invariant three-month process. Intensive longitudinal and experimental designs are needed for stronger causal interpretation (Dormann and Griffin, 2015; Hives et al., 2025; Rohrer and Murayama, 2023).

The study was not prospectively registered, and the internal analysis plan was neither publicly registered nor independently time-stamped. The post-data design-sensitivity simulation characterizes the performance of the completed design and is not an *a priori* power analysis. The sample was limited to adult undergraduates from six institutions in Chengdu, and the analyses focused on global PSU and EA scores. Dimension-specific associations, mediators, and moderators remain to be examined. Future research should measure time-varying confounders and candidate mechanisms while preserving the distinction between between-person and within-person processes.

## 6 Conclusion

This four-wave RI-CLPM study found that within-person elevations in self-reported exercise-adherence tendency were prospectively associated with lower subsequent problematic smartphone use, whereas the corresponding PSU-to-EA paths were not statistically significant. The directional difference was significant in the first two intervals but not from T3 to T4, and the broad pattern was reproduced across three standardizations and multiple sensitivity analyses. The between-person association was measurement dependent. Experimental and intensive longitudinal research is needed before concluding that changing exercise adherence would reduce problematic smartphone use.

## Data Availability

The de-identified item-level dataset, prorated scale-score dataset, demographic codebook, and Supplementary Tables S1–S14 are included in the Supplementary Material. Raw linkage information and materials that could permit re-identification are not publicly available because participant confidentiality must be protected and the broader longitudinal follow-up is ongoing. Further inquiries may be directed to the corresponding author.
