# Supplementary material for: Within-person reciprocal associations between problematic smartphone use and exercise adherence among university students: a four-wave random-intercept cross-lagged panel study
Source: Front Psychol. 2026 Aug 24;17:1907886. doi: 10.3389/fpsyg.2026.1907886 (PMC13547006; doi:10.3389/fpsyg.2026.1907886)
Supplement: Supplementary file 1 [file Table_1.DOCX]

**Supplementary Tables**

*Within-Person Reciprocal Associations Between Problematic Smartphone Use and Exercise Adherence Among University Students: A Four-Wave Random-Intercept Cross-Lagged Panel Study*

**Table S1. Exploratory multivariable logistic regressions predicting wave-level absence of both focal outcomes**

| **Predictor** | **Coding** | **Neither PSU nor EA valid at ≥1 follow-up wave OR [95% CI]** | **p** | **Neither PSU nor EA valid at T4 OR [95% CI]** | **p** |
| --- | --- | --- | --- | --- | --- |
| Baseline PSU | Per 1-SD increase | 1.179 [1.026, 1.354] | .020 | 1.142 [.967, 1.348] | .117 |
| Baseline EA | Per 1-SD increase | 1.199 [1.042, 1.378] | .011 | 1.216 [1.026, 1.441] | .024 |
| Female | Versus male | 1.172 [.892, 1.541] | .255 | 1.176 [.844, 1.639] | .337 |
| Other/prefer not to say | Versus male (n = 6; sparse) | NE | — | NE | — |
| Age | Older category | 1.144 [1.021, 1.282] | .021 | 1.108 [.966, 1.272] | .142 |
| Subjective SES | Higher category | .788 [.647, .961] | .019 | .769 [.605, .977] | .032 |
| BMI category | Ordered trend^†^ | 1.267 [1.035, 1.550] | .022 | 1.349 [1.061, 1.713] | .014 |
| Self-rated health | Better self-rated health category | 1.275 [1.064, 1.528] | .008 | 1.331 [1.068, 1.658] | .011 |
| Injury/exercise limitation | Greater severity | 1.290 [.962, 1.730] | .089 | 1.335 [.953, 1.870] | .092 |
| Organized exercise frequency | Higher frequency | 1.212 [1.046, 1.405] | .011 | 1.195 [1.001, 1.427] | .049 |
| Daily smartphone-use duration | Longer-duration category | 1.019 [.892, 1.164] | .784 | 1.032 [.879, 1.212] | .699 |
| Exercise habit | Stronger-habit category | 1.248 [1.069, 1.458] | .005 | 1.217 [1.011, 1.466] | .038 |

**Note.** PSU = problematic smartphone use; EA = exercise adherence; SES = socioeconomic status; BMI = body mass index; OR = odds ratio; NE = not estimable. All predictors were entered simultaneously. The first model defined an event as having neither a valid PSU score nor a valid EA score at one or more follow-up waves (N = 1,241; 287 events). The second model used the same wave-level definition at T4 (N = 1,241; 174 events). These outcomes differ from the complete-case criterion, which required valid PSU and EA scores at all four waves and yielded n = 953. Ordered predictors were scored in the following directions: age, 18 < 19 < 20 < 21 < ≥22 years; subjective SES, low < middle < high; BMI, underweight < normal weight < overweight < obesity; self-rated health, poor < fair < good < very good; injury/exercise limitation, none < minor < obvious; organized exercise frequency, none < once per week < twice per week < three or more times per week; daily smartphone-use duration, ≤2 < 2–4 < 4–6 < 6–8 < >8 hours per day; and exercise habit, none < occasional < relatively stable < regular training. Thus, an OR above 1.0 for self-rated health denotes greater odds of wave-level nonparticipation at better self-rated-health categories. These ordered predictors were entered as single linear trend terms for an exploratory missingness screen. ^†^The BMI trend should not be interpreted as an equal linear risk increment across clinically distinct categories or as a substitute for category-specific contrasts. The other/prefer-not-to-say gender category contained six participants and produced sparse-category separation under ordinary maximum likelihood; no inference was drawn from its non-estimable coefficient. P values are nominal, two-sided, and unadjusted for multiple comparisons. These models identify observed predictors of missingness but do not verify the MAR assumption.

**Table S2. Baseline comparison of the theoretical multidimensional structure with a single-factor alternative**

| **Model** | **χ² (df)** | **p** | **CFI** | **TLI** | **RMSEA [90% CI]** | **SRMR** | **AIC** | **BIC** |
| --- | --- | --- | --- | --- | --- | --- | --- | --- |
| Theoretical six-factor model | 204.475 (237) | .938 | 1.000 | 1.004 | .000 [.000, .003] | .016 | 91,749.568 | 92,195.327 |
| Single-factor model | 5,470.944 (252) | < .001 | .481 | .431 | .129 [.126, .132] | .143 | 97,049.978 | 97,418.882 |

**Note.** The six-factor model specified the three PSU dimensions and three EA dimensions as correlated substantive factors. The single-factor alternative loaded all 24 items on one factor. The poor fit of the single-factor alternative indicates that one general factor was insufficient to represent the item covariance; it does not demonstrate absence of common method bias. Fit-index values above 1.0 are reported as produced by Mplus.

**Table S3. Sensitivity analyses using complete cases, partial equality constraints, and a multiple-indicator latent RI-CLPM**

**Panel A. Model fit**

| **Model** | **N** | **χ² (df)** | **p** | **CFI** | **TLI** | **RMSEA [90% CI]** | **SRMR** |
| --- | --- | --- | --- | --- | --- | --- | --- |
| Primary observed-score RI-CLPM with FIML | 1,241 | 12.320 (9) | .196 | .999 | .996 | .017 [.000, .039] | .016 |
| Complete-case observed-score RI-CLPM | 953 | 15.495 (9) | .078 | .997 | .990 | .028 [.000, .050] | .019 |
| Post hoc partial-equality RI-CLPM | 1,241 | 18.894 (13) | .126 | .997 | .994 | .019 [.000, .037] | .021 |
| Multiple-indicator latent RI-CLPM | 1,241 | 274.395 (221) | .008 | .994 | .993 | .014 [.007, .019] | .026 |

**Panel B. Direct STDYX focal estimates**

| **Path** | **Primary FIML β/r [95% CI], p** | **Complete-case β/r [95% CI], p** | **Partial-equality β/r [95% CI], p** | **Latent RI-CLPM β/r [95% CI], p** |
| --- | --- | --- | --- | --- |
| EA1 → PSU2 | −.293 [−.373, −.213], < .001 | −.297 [−.381, −.212], < .001 | −.308 [−.385, −.231], < .001 | −.478 [−.619, −.338], < .001 |
| EA2 → PSU3 | −.185 [−.271, −.099], < .001 | −.162 [−.253, −.070], .001 | −.182 [−.269, −.095], < .001 | −.314 [−.458, −.169], < .001 |
| EA3 → PSU4 | −.136 [−.208, −.064], < .001 | −.146 [−.220, −.072], < .001 | −.142 [−.214, −.071], < .001 | −.167 [−.273, −.061], .002 |
| PSU1 → EA2 | .017 [−.067, .102], .687 | .032 [−.060, .125], .494 | −.014 [−.073, .046], .651 | .016 [−.132, .164], .831 |
| PSU2 → EA3 | .027 [−.059, .112], .540 | .027 [−.062, .116], .547 | −.013 [−.068, .042], .651 | .078 [−.069, .225], .296 |
| PSU3 → EA4 | −.064 [−.142, .014], .106 | −.055 [−.137, .028], .195 | −.013 [−.071, .045], .653 | −.086 [−.207, .035], .163 |
| RI_PSU ↔ RI_EA | −.220 [−.348, −.092], .001 | −.223 [−.359, −.087], .001 | −.204 [−.336, −.072], .002 | −.091 [−.336, .154], .466 |

**Note.** RI-CLPM = random-intercept cross-lagged panel model; PSU = problematic smartphone use; EA = exercise adherence; RI = random intercept; FIML = full-information maximum likelihood. The complete-case model required valid PSU and EA scores at all four waves. The partial-equality model constrained the three PSU-to-EA paths and the three EA autoregressive paths to equality. The multiple-indicator latent model used the three theoretically defined subscale means as indicators of each construct at every wave and estimated measurement error explicitly. Equality constraints on unstandardized paths can yield slightly different standardized coefficients across intervals.

**Table S4. Robustness of the cross-lagged findings across alternative covariate specifications**

| **Model** | **Covariate specification** | **Maximum \|Δβ\| relative to M1** | **All EA→PSU paths significant** | **All PSU→EA paths nonsignificant** | **Estimation acceptable** |
| --- | --- | --- | --- | --- | --- |
| Unadjusted M1 | No baseline covariates | .000 | Yes | Yes | Yes |
| Core covariates | Gender, age, SES, household registration, ethnicity, BMI, health, and injury predicting both random intercepts | .004 | Yes | Yes | Yes |
| Core + academic/context | Core covariates plus major, university type, and region | .003 | Yes | Yes | Yes |
| Core + proximal behaviors | Core covariates plus smartphone-use duration, exercise habit, and exercise frequency | .007 | Yes | Yes | Yes |
| Core + T1 within adjustment | Core covariates predicting both random intercepts and T1 within-person deviation factors | .007 | Yes | Yes | Yes |

**Note.** Δβ is the absolute difference between an adjusted direct STDYX cross-lagged coefficient and the corresponding coefficient from the unadjusted M1 model. All covariate-related Mplus models terminated normally without warnings or errors. The selected covariates were complete for all 1,241 participants, the maximum variance inflation factor was 2.18, and all estimated latent variances were positive.

**Table S5. Coding of covariates used in the sensitivity analyses**

| **Covariate set** | **Variable** | **Dummy-coded categories** | **Reference category** |
| --- | --- | --- | --- |
| Core | Gender | Female; other/prefer not to report | Male |
| Core | Age | 19 years; 20 years; 21 years; 22 years or older | 18 years |
| Core | Subjective SES | Low; high | Middle |
| Core | Household registration | Rural | Urban |
| Core | Ethnicity | Ethnic minority | Han |
| Core | BMI category | Underweight; overweight; obesity | Normal weight |
| Core | Self-rated health | Poor; fair; very good | Good |
| Core | Injury status | Minor; obvious | None |
| Academic/context | Major | Humanities/social sciences/education; economics/management; medicine/health; arts; physical education; other | STEM |
| Academic/context | University type | STEM-oriented; applied; specialized | Comprehensive |
| Academic/context | Region | Central China; Western China; Northeastern China | Eastern China |
| Proximal behaviors | Daily smartphone use | ≤2 hours; 2–4 hours; 6–8 hours; >8 hours | 4–6 hours |
| Proximal behaviors | Exercise habit | Occasional exercise; relatively stable habit; regular training | None |
| Proximal behaviors | Organized exercise frequency | Once per week; twice per week; three or more times per week | None |

**Note.** SES = socioeconomic status; BMI = body mass index; STEM = science, technology, engineering, and mathematics. All covariates were measured at baseline. In the preferred core model, the dummy indicators predicted the PSU and EA random intercepts. The alternate-placement sensitivity model additionally allowed the core covariates to predict the T1 within-person deviation factors. The 177-participant university-type category was coded as Applied university.

**Table S6. Component-specific equality tests and the post hoc partial-equality RI-CLPM**

| **Model** | **Equality constraints** | **χ² (df)** | **p** | **CFI** | **TLI** | **RMSEA [90% CI]** | **SRMR** | **AIC** | **BIC** | **Robust Δχ² (df) vs. M1** | **p** |
| --- | --- | --- | --- | --- | --- | --- | --- | --- | --- | --- | --- |
| M5 | PSU-to-EA paths equal | 15.647 (11) | .155 | .998 | .995 | .018 [.000, .038] | .017 | 21,707.582 | 21,876.663 | 3.324 (2) | .190 |
| M6 | EA-to-PSU paths equal | 20.790 (11) | .036 | .996 | .989 | .027 [.007, .044] | .023 | 21,712.678 | 21,881.759 | 8.483 (2) | .014 |
| M7 | EA autoregressive paths equal | 15.629 (11) | .156 | .998 | .995 | .018 [.000, .038] | .020 | 21,707.276 | 21,876.357 | 3.358 (2) | .187 |
| M8 | PSU autoregressive paths equal | 30.642 (11) | .001 | .991 | .978 | .038 [.022, .054] | .031 | 21,721.614 | 21,890.695 | 20.753 (2) | < .001 |
| M9ᵃ | PSU-to-EA and EA autoregressive paths equal | 18.894 (13) | .126 | .997 | .994 | .019 [.000, .037] | .021 | 21,706.508 | 21,865.342 | 6.614 (4) | .158 |

**Note.** RI-CLPM = random-intercept cross-lagged panel model; AIC = Akaike information criterion; BIC = Bayesian information criterion. Robust scaled chi-square difference tests compare each model with the fully unconstrained M1. ᵃM9 combined the equality constraints supported in M5 and M7 and was treated as a post hoc sensitivity specification.

**Table S7. Directional comparisons across three standardization methods**

| **Standardization** | **Interval** | **EA→PSU** | **PSU→EA** | **Difference** | **SE** | **95% CI** | **p** |
| --- | --- | --- | --- | --- | --- | --- | --- |
| Time-specific within-person | T1-T2 | −.293 | .017 | −.310 | .054 | [−.417, −.204] | < .001 |
| Time-specific within-person | T2-T3 | −.185 | .027 | −.212 | .057 | [−.323, −.100] | < .001 |
| Time-specific within-person | T3-T4 | −.136 | −.064 | −.072 | .051 | [−.173, .029] | .161 |
| Time-specific within-person | Joint | — | — | χ²(3) = 59.159 | — | — | < .001 |
| Pooled within-person | T1-T2 | −.282 | .017 | −.299 | .052 | [−.401, −.197] | < .001 |
| Pooled within-person | T2-T3 | −.189 | .028 | −.218 | .060 | [−.334, −.101] | < .001 |
| Pooled within-person | T3-T4 | −.136 | −.064 | −.072 | .052 | [−.173, .030] | .165 |
| Pooled within-person | Joint | — | — | χ²(3) = 59.258 | — | — | < .001 |
| Pooled observed total-score SD | T1-T2 | −.264 | .018 | −.282 | .052 | [−.384, −.180] | < .001 |
| Pooled observed total-score SD | T2-T3 | −.177 | .030 | −.208 | .060 | [−.324, −.091] | < .001 |
| Pooled observed total-score SD | T3-T4 | −.127 | −.069 | −.059 | .052 | [−.161, .043] | .260 |
| Pooled observed total-score SD | Joint | — | — | χ²(3) = 54.469 | — | — | < .001 |

**Note.** Difference = EA-to-PSU minus PSU-to-EA. Time-specific within-person standardization is the primary comparison; pooled within-person and pooled observed-total-score SD methods are sensitivity checks. All three methods supported an overall difference and significant interval differences at T1–T2 and T2–T3, but not at T3–T4. The time-specific within-person joint Wald test was χ²(3) = 59.159, p < .001.

**Table S8. Exercise-adherence score distributions and exercise-habit sensitivity analyses**

**Panel A. EA distributions by baseline exercise-habit category**

| **Wave** | **Baseline habit** | **Valid n** | **M** | **SD** | **Median** | **IQR** | **Floor %** | **Near-floor %** | **Ceiling %** |
| --- | --- | --- | --- | --- | --- | --- | --- | --- | --- |
| T1 | No exercise | 457 | 3.438 | 0.778 | 3.500 | [2.929, 4.000] | 0.00 | 1.09 | 0.66 |
| T1 | Occasional exercise | 452 | 3.422 | 0.771 | 3.429 | [2.929, 4.000] | 0.00 | 0.00 | 0.88 |
| T1 | Relatively stable habit | 288 | 3.421 | 0.784 | 3.429 | [2.857, 4.000] | 0.00 | 1.39 | 0.69 |
| T1 | Regular training | 44 | 3.525 | 0.789 | 3.679 | [3.018, 4.161] | 0.00 | 0.00 | 0.00 |
| T2 | No exercise | 428 | 3.390 | 0.756 | 3.429 | [2.907, 3.929] | 0.00 | 0.70 | 0.00 |
| T2 | Occasional exercise | 415 | 3.504 | 0.768 | 3.571 | [2.929, 4.071] | 0.00 | 1.20 | 0.00 |
| T2 | Relatively stable habit | 269 | 3.543 | 0.773 | 3.571 | [3.071, 4.071] | 0.37 | 0.74 | 0.74 |
| T2 | Regular training | 40 | 3.518 | 0.708 | 3.536 | [3.054, 4.054] | 0.00 | 0.00 | 0.00 |
| T3 | No exercise | 416 | 3.368 | 0.828 | 3.429 | [2.786, 4.000] | 0.00 | 0.72 | 0.72 |
| T3 | Occasional exercise | 415 | 3.400 | 0.779 | 3.429 | [2.929, 4.000] | 0.00 | 0.72 | 0.00 |
| T3 | Relatively stable habit | 252 | 3.480 | 0.762 | 3.500 | [2.929, 4.083] | 0.00 | 0.40 | 0.00 |
| T3 | Regular training | 39 | 3.474 | 0.840 | 3.429 | [2.857, 4.146] | 0.00 | 0.00 | 0.00 |
| T4 | No exercise | 403 | 3.397 | 0.801 | 3.429 | [2.810, 4.000] | 0.00 | 0.99 | 0.50 |
| T4 | Occasional exercise | 387 | 3.475 | 0.768 | 3.538 | [2.926, 4.071] | 0.00 | 0.00 | 0.00 |
| T4 | Relatively stable habit | 238 | 3.472 | 0.766 | 3.519 | [2.857, 4.143] | 0.00 | 0.42 | 0.00 |
| T4 | Regular training | 38 | 3.556 | 0.820 | 3.577 | [3.000, 4.214] | 0.00 | 0.00 | 2.63 |

**Panel B. Exerciser-only RI-CLPM focal estimates (baseline any exercise; n = 784)**

| **Path** | **Interval** | **β** | **SE** | **95% CI** | **p** |
| --- | --- | --- | --- | --- | --- |
| EA T1 -> PSU T2 | T1-T2 | −.307 | .051 | [−.406, −.208] | < .001 |
| PSU T1 -> EA T2 | T1-T2 | .003 | .055 | [−.104, .110] | .952 |
| EA T2 -> PSU T3 | T2-T3 | −.151 | .057 | [−.263, −.038] | .008 |
| PSU T2 -> EA T3 | T2-T3 | .006 | .057 | [−.106, .117] | .917 |
| EA T3 -> PSU T4 | T3-T4 | −.156 | .045 | [−.244, −.067] | .001 |
| PSU T3 -> EA T4 | T3-T4 | −.086 | .055 | [−.193, .021] | .117 |

**Panel C. None-versus-any-exercise multigroup tests**

| **Test** | **Direction** | **Robust Δχ²** | **df** | **p** |
| --- | --- | --- | --- | --- |
| All six cross-lagged paths equal across groups | Both directions | 4.059 | 6 | .669 |
| Three EA-to-PSU paths equal across groups | EA -> PSU | 2.769 | 3 | .429 |
| Three PSU-to-EA paths equal across groups | PSU -> EA | 0.877 | 3 | .831 |

**Note.** EA = exercise adherence; PSU = problematic smartphone use. Near-floor was defined as EA ≤ 1.50. No separate not-applicable response code was present. In Panel B, all three EA-to-PSU paths remained negative and significant, all PSU-to-EA paths remained nonsignificant, and the maximum |Δβ| from the full-sample M1 was .034. The exerciser-only model fit was χ²(9) = 9.651, p = .380, CFI = 1.000, TLI = .999, RMSEA = .010, 90% CI [.000, .042], and SRMR = .019. Distributional similarity and sensitivity-model agreement do not establish content validity among non-exercisers.

**Table S9. Auxiliary-variable FIML sensitivity model**

| **Path** | **Interval** | **β/r** | **SE** | **95% CI** | **p** |
| --- | --- | --- | --- | --- | --- |
| EA T1 -> PSU T2 | T1-T2 | −.292 | .041 | [−.372, −.212] | < .001 |
| PSU T1 -> EA T2 | T1-T2 | .015 | .043 | [−.069, .100] | .722 |
| EA T2 -> PSU T3 | T2-T3 | −.187 | .044 | [−.273, −.101] | < .001 |
| PSU T2 -> EA T3 | T2-T3 | .027 | .043 | [−.058, .112] | .535 |
| EA T3 -> PSU T4 | T3-T4 | −.136 | .037 | [−.209, −.064] | < .001 |
| PSU T3 -> EA T4 | T3-T4 | −.066 | .039 | [−.144, .011] | .094 |
| RI_PSU WITH RI_EA | Between-person | −.220 | .066 | [−.349, −.092] | .001 |

**Note.** The simplified auxiliary-variable FIML model incorporated gender, age, subjective SES, BMI category, self-rated health, injury status, organized exercise frequency, daily smartphone-use duration, and exercise habit. Model fit was χ²(9) = 12.329, p = .195, CFI = .999, TLI = .995, RMSEA = .017, 90% CI [.000, .039], and SRMR = .016. The maximum absolute change in a cross-lagged STDYX estimate relative to primary M1 was .002. The model terminated normally with positive latent variances and no |r| > 1, but retained a nonpositive-definite PSI warning; it is therefore interpreted as a qualified sensitivity result rather than validation of MAR.

**Table S10. Delta-adjustment sensitivity to specified departures from MAR**

**Panel A. Selected multiple-imputation delta scenarios**

| **ΔPSU / ΔEA** | **Path** | **b** | **SE** | **95% CI** | **p** |
| --- | --- | --- | --- | --- | --- |
| 0 SD / 0 SD | PSU T1 -> EA T2 | .015 | .033 | [−.050, .079] | .651 |
| 0 SD / 0 SD | PSU T2 -> EA T3 | .026 | .037 | [−.045, .098] | .471 |
| 0 SD / 0 SD | PSU T3 -> EA T4 | −.051 | .031 | [−.113, .010] | .103 |
| 0 SD / 0 SD | EA T1 -> PSU T2 | −.345 | .049 | [−.441, −.248] | < .001 |
| 0 SD / 0 SD | EA T2 -> PSU T3 | −.225 | .056 | [−.334, −.116] | < .001 |
| 0 SD / 0 SD | EA T3 -> PSU T4 | −.163 | .047 | [−.254, −.071] | .001 |
| −0.50 SD / −0.50 SD | PSU T1 -> EA T2 | .015 | .033 | [−.050, .080] | .649 |
| −0.50 SD / −0.50 SD | PSU T2 -> EA T3 | .037 | .036 | [−.033, .107] | .302 |
| −0.50 SD / −0.50 SD | PSU T3 -> EA T4 | −.051 | .031 | [−.112, .009] | .097 |
| −0.50 SD / −0.50 SD | EA T1 -> PSU T2 | −.348 | .050 | [−.447, −.250] | < .001 |
| −0.50 SD / −0.50 SD | EA T2 -> PSU T3 | −.224 | .056 | [−.333, −.115] | < .001 |
| −0.50 SD / −0.50 SD | EA T3 -> PSU T4 | −.138 | .047 | [−.230, −.047] | .003 |
| −1.00 SD / −1.00 SD | PSU T1 -> EA T2 | .013 | .034 | [−.053, .079] | .703 |
| −1.00 SD / −1.00 SD | PSU T2 -> EA T3 | .041 | .035 | [−.027, .109] | .241 |
| −1.00 SD / −1.00 SD | PSU T3 -> EA T4 | −.044 | .031 | [−.104, .016] | .146 |
| −1.00 SD / −1.00 SD | EA T1 -> PSU T2 | −.356 | .053 | [−.460, −.253] | < .001 |
| −1.00 SD / −1.00 SD | EA T2 -> PSU T3 | −.213 | .055 | [−.322, −.105] | < .001 |
| −1.00 SD / −1.00 SD | EA T3 -> PSU T4 | −.107 | .047 | [−.199, −.016] | .021 |

**Panel B. Tipping-point summary for EA-to-PSU paths**

| **Path** | **Tipping point found** | **Maximum tested** | **Adverse direction** | **Conclusion** |
| --- | --- | --- | --- | --- |
| EA T1 -> PSU T2 | No | 1.00 SD | Joint negative shift in PSU and EA | No loss of negative statistical significance among completed scenarios |
| EA T2 -> PSU T3 | No | 1.00 SD | Joint negative shift in PSU and EA | No loss of negative statistical significance among completed scenarios |
| EA T3 -> PSU T4 | No | 1.00 SD | Joint negative shift in PSU and EA | No loss of negative statistical significance among completed scenarios |

**Note.** Fifty imputed datasets were generated with predictive mean matching, 20 iterations, and seed 20260723. Delta shifts were applied only to originally missing values and scores were constrained to their theoretical ranges. These scenarios evaluate sensitivity to specified departures from MAR; they do not identify the true missingness mechanism. At the joint −1 SD/−1 SD boundary, the weakest EA-to-PSU path was EA T3→PSU T4, b = −.107, 95% CI [−.199, −.016], p = .021.

**Table S11. Multidimensional measurement-model fit and reliability**

**Panel A. Correlated three-factor CFA fit by construct and wave**

| **Construct** | **Wave** | **N** | **χ² (df)** | **p** | **CFI** | **TLI** | **RMSEA** | **90% CI** | **SRMR** |
| --- | --- | --- | --- | --- | --- | --- | --- | --- | --- |
| PSU | T1 | 1241 | 27.299 (32) | .704 | 1.000 | 1.002 | 0.000 | [0.000, 0.017] | 0.013 |
| PSU | T2 | 1152 | 28.221 (32) | .658 | 1.000 | 1.001 | 0.000 | [0.000, 0.018] | 0.012 |
| PSU | T3 | 1122 | 59.785 (32) | .002 | 0.993 | 0.990 | 0.028 | [0.017, 0.039] | 0.017 |
| PSU | T4 | 1067 | 28.967 (32) | .621 | 1.000 | 1.001 | 0.000 | [0.000, 0.020] | 0.012 |
| EA | T1 | 1241 | 62.767 (74) | .821 | 1.000 | 1.003 | 0.000 | [0.000, 0.010] | 0.015 |
| EA | T2 | 1152 | 76.210 (74) | .407 | 1.000 | 0.999 | 0.005 | [0.000, 0.018] | 0.017 |
| EA | T3 | 1122 | 87.696 (74) | .132 | 0.997 | 0.997 | 0.013 | [0.000, 0.022] | 0.018 |
| EA | T4 | 1067 | 60.323 (74) | .874 | 1.000 | 1.004 | 0.000 | [0.000, 0.009] | 0.014 |

**Panel B. Total-score reliability**

| **Construct** | **Wave** | **α** | **ω total** | **95% CI** | **ω hierarchical** | **95% CI** |
| --- | --- | --- | --- | --- | --- | --- |
| PSU | T1 | 0.875 | 0.901 | [0.893, 0.909] | 0.784 | [0.763, 0.805] |
| PSU | T2 | 0.869 | 0.895 | [0.886, 0.903] | 0.786 | [0.765, 0.807] |
| PSU | T3 | 0.874 | 0.899 | [0.890, 0.908] | 0.790 | [0.767, 0.812] |
| PSU | T4 | 0.876 | 0.901 | [0.892, 0.910] | 0.789 | [0.767, 0.812] |
| EA | T1 | 0.879 | 0.900 | [0.892, 0.909] | 0.768 | [0.745, 0.791] |
| EA | T2 | 0.875 | 0.898 | [0.889, 0.906] | 0.759 | [0.734, 0.784] |
| EA | T3 | 0.885 | 0.905 | [0.897, 0.913] | 0.776 | [0.753, 0.800] |
| EA | T4 | 0.878 | 0.897 | [0.889, 0.906] | 0.779 | [0.757, 0.802] |

**Panel C. Subscale model-based omega**

| **Construct** | **Subscale** | **T1** | **T2** | **T3** | **T4** |
| --- | --- | --- | --- | --- | --- |
| PSU | tolerance | 0.807 | 0.784 | 0.777 | 0.775 |
| PSU | withdrawal | 0.780 | 0.775 | 0.813 | 0.847 |
| PSU | negative effect | 0.783 | 0.777 | 0.774 | 0.760 |
| EA | behavioral habit | 0.796 | 0.799 | 0.801 | 0.794 |
| EA | effort input | 0.817 | 0.811 | 0.823 | 0.799 |
| EA | emotional experience | 0.776 | 0.772 | 0.789 | 0.772 |

**Note.** Omega total was calculated for the unit-weighted total score from the correlated three-factor model. Omega hierarchical is supplementary and does not imply unidimensionality. All selected three-factor models terminated normally without material warnings or improper solutions.

**Table S12. Within-wave covariances and within-person variances from primary M1**

**Panel A. Within-wave PSU–EA covariances**

| **Wave** | **b** | **SEb** | **95% CI for b** | **r** | **SEr** | **95% CI for r** | **p** |
| --- | --- | --- | --- | --- | --- | --- | --- |
| T1 | −.102 | .024 | [−.149, −.055] | −.186 | .041 | [−.266, −.106] | < .001 |
| T2 | −.072 | .019 | [−.110, −.034] | −.157 | .041 | [−.239, −.076] | < .001 |
| T3 | −.071 | .020 | [−.110, −.032] | −.141 | .038 | [−.216, −.066] | < .001 |
| T4 | −.060 | .018 | [−.095, −.026] | −.124 | .036 | [−.194, −.054] | .001 |

**Panel B. Within-person variance parameters**

| **Type** | **Wave** | **Parameter** | **Variance** | **SE** | **95% CI** |
| --- | --- | --- | --- | --- | --- |
| Baseline within-person variance | T1 | WX1 | 0.694 | .037 | [.621, .766] |
| Baseline within-person variance | T1 | WY1 | 0.431 | .024 | [.384, .478] |
| Within-person innovation/residual variance | T2 | WX2 | 0.538 | .032 | [.476, .601] |
| Within-person innovation/residual variance | T2 | WY2 | 0.388 | .020 | [.348, .428] |
| Within-person innovation/residual variance | T3 | WX3 | 0.612 | .032 | [.549, .674] |
| Within-person innovation/residual variance | T3 | WY3 | 0.417 | .019 | [.380, .454] |
| Within-person innovation/residual variance | T4 | WX4 | 0.595 | .028 | [.540, .651] |
| Within-person innovation/residual variance | T4 | WY4 | 0.399 | .019 | [.362, .436] |

**Note.** Within-wave covariances were omitted from Figure 3 for readability. T2–T4 variance rows are innovation/residual variances and should not be interpreted as total model-implied within-person variances at those waves.

**Table S13. Post-data Monte Carlo design-sensitivity analysis**

| **Scenario** | **EA→PSU 1** | **EA→PSU 2** | **EA→PSU 3** | **PSU→EA 1–3** | **Detect 1** | **Detect 2** | **Detect 3** | **Joint Wald** | **Rep.** | **Conv.** |
| --- | --- | --- | --- | --- | --- | --- | --- | --- | --- | --- |
| A: EA −.05; reverse 0 | −.05 | −.05 | −.05 | 0, 0, 0 | 0.212 | 0.202 | 0.244 | 0.354 | 500 | 1.000 |
| A: EA −.08; reverse 0 | −.08 | −.08 | −.08 | 0, 0, 0 | 0.503 | 0.467 | 0.555 | 0.791 | 1000 | 1.000 |
| A: EA −.10; reverse 0 | −.10 | −.10 | −.10 | 0, 0, 0 | 0.684 | 0.634 | 0.783 | 0.955 | 1000 | 1.000 |
| A: EA −.15; reverse 0 | −.15 | −.15 | −.15 | 0, 0, 0 | 0.955 | 0.915 | 0.984 | 0.999 | 1000 | 1.000 |
| A: EA −.20; reverse 0 | −.20 | −.20 | −.20 | 0, 0, 0 | 0.996 | 0.994 | 1.000 | 1.000 | 500 | 1.000 |
| C: time-varying | −.30 | −.18 | −.14 | 0, 0, −.06 | 1.000 | 0.992 | 0.964 | 1.000 | 500 | 1.000 |

**Note.** This simulation was conducted after data collection as a design-sensitivity analysis and was not an a priori or observed-power calculation. N = 1,241, MLR, and the aggregate empirical eight-score missing-pattern distribution were used. Across formal scenarios, convergence was 1.000; maximum absolute bias was .0048, maximum absolute relative standard-error bias was .0701, and 95% coverage ranged from .928 to .966. Scenario C used rounded values close to the empirical path pattern.

**Table S14. Model diagnostics and supplementary-data index**

**Panel A. Key model diagnostics**

| **Model family** | **Termination** | **Warning** | **Diagnostic summary** | **Role** |
| --- | --- | --- | --- | --- |
| Primary observed-score M1 | Normal | None | No negative variance or \|r\| > 1 | Primary inference |
| Time-specific WP directional model | Normal | None | MODEL CONSTRAINT estimates matched direct STDYX | Primary direction test |
| Multiple-indicator latent RI-CLPM | Normal | None | No improper solution | Measurement sensitivity |
| Exerciser-only RI-CLPM | Normal | None | No improper solution | EA sensitivity |
| Exercise-habit multigroup models | Normal | None | Final five models admissible | Effect-modification sensitivity |
| Auxiliary-variable FIML | Normal | PSI not positive definite | No negative free variance or \|r\| > 1 | Qualified missing-data sensitivity |
| Delta-adjusted MI models | Normal | None | 50/50 imputations completed in selected models | MNAR sensitivity |
| Three-factor reliability CFAs | Normal | None | Eight selected models admissible | Reliability |

**Panel B. Supplementary data files**

| **Material** | **Contents** |
| --- | --- |
| De-identified item-level data | Coded baseline variables and four-wave PSU and EA item responses. |
| Prorated scale-score data | Coded baseline variables and four-wave PSU and EA subscale and total mean scores based on the 80% item-completion rule. |
| Demographic codebook | Category labels, frequencies, and percentages for coded baseline variables. |

**Note.** The supplementary data files contain de-identified analytic data and a demographic codebook; they contain no names, mobile-phone numbers, student numbers, participant-level university identifiers, or linkage codes. Raw linkage information and any materials that could permit re-identification are not shared.
